# A methodological systematic review of what’s wrong with meta-ethnography reporting

**DOI:** 10.1186/1471-2288-14-119

**Published:** 2014-11-19

**Authors:** Emma F France, Nicola Ring, Rebecca Thomas, Jane Noyes, Margaret Maxwell, Ruth Jepson

**Affiliations:** Nursing Midwifery and Allied Health Professions Research Unit, University of Stirling and Glasgow Caledonian University, Unit 13 Scion House, Stirling University Innovation Park, Stirling, FK9 4NF Scotland, UK; School of Health Sciences, University of Stirling, Stirling, FK9 4LA Scotland, UK; School of Social Sciences, Bangor University, Bangor, Gwynedd, LL57 2DG UK; Scottish Collaboration for Public Health Research and Policy (SCPHRP), University of Edinburgh, 20 West Richmond Street, Edinburgh, EH8 9DX Scotland, UK

**Keywords:** Meta-ethnography, Systematic review, Qualitative health research, Reporting, Qualitative synthesis, Health, Evidence-based practice

## Abstract

**Background:**

Syntheses of qualitative studies can inform health policy, services and our understanding of patient experience. Meta-ethnography is a systematic seven-phase interpretive qualitative synthesis approach well-suited to producing new theories and conceptual models. However, there are concerns about the quality of meta-ethnography reporting, particularly the analysis and synthesis processes. Our aim was to investigate the application and reporting of methods in recent meta-ethnography journal papers, focusing on the analysis and synthesis process and output.

**Methods:**

Methodological systematic review of health-related meta-ethnography journal papers published from 2012–2013. We searched six electronic databases, Google Scholar and Zetoc for papers using key terms including ‘meta-ethnography.’ Two authors independently screened papers by title and abstract with 100% agreement. We identified 32 relevant papers. Three authors independently extracted data and all authors analysed the application and reporting of methods using content analysis.

**Results:**

Meta-ethnography was applied in diverse ways, sometimes inappropriately. In 13% of papers the approach did not suit the research aim. In 66% of papers reviewers did not follow the principles of meta-ethnography. The analytical and synthesis processes were poorly reported overall. In only 31% of papers reviewers clearly described how they analysed conceptual data from primary studies (phase 5, ‘translation’ of studies) and in only one paper (3%) reviewers explicitly described how they conducted the analytic synthesis process (phase 6). In 38% of papers we could not ascertain if reviewers had achieved any new interpretation of primary studies. In over 30% of papers seminal methodological texts which could have informed methods were not cited.

**Conclusions:**

We believe this is the first in-depth methodological systematic review of meta-ethnography conduct and reporting. Meta-ethnography is an evolving approach. Current reporting of methods, analysis and synthesis lacks clarity and comprehensiveness. This is a major barrier to use of meta-ethnography findings that could contribute significantly to the evidence base because it makes judging their rigour and credibility difficult. To realise the high potential value of meta-ethnography for enhancing health care and understanding patient experience requires reporting that clearly conveys the methodology, analysis and findings. Tailored meta-ethnography reporting guidelines, developed through expert consensus, could improve reporting.

**Electronic supplementary material:**

The online version of this article (doi:10.1186/1471-2288-14-119) contains supplementary material, which is available to authorized users.

## Background

Evidence-based health care requires robust, synthesised evidence of all types in combination with clinical judgment and information on patient preferences. Quantitative evidence syntheses are now routinely used internationally to inform clinical guidelines, health technology assessment and intervention development [1]. The synthesis of qualitative studies - qualitative evidence synthesis (QES) - is becoming more common in health-related research [2] and increasingly recognised as important for informing health care policy and practice [1, 3]. While quantitative syntheses can provide evidence of intervention effectiveness, qualitative syntheses can show intervention feasibility, appropriateness and acceptability to patients and thus inform intervention implementation [4]. Qualitative evidence syntheses can advance understanding of complex health care issues by developing theory about how a health service, policy, strategy, programme or intervention works or not and how it impacts on patient experience [3]. They can also be used to advance understanding of illness experiences across a broad spectrum of illnesses for different population groups across the life course or illness trajectory [5, 6]. High quality meta-ethnographies [5] have informed recent clinical guidelines [7].

There are many possible approaches that can be used to synthesise qualitative research [8–10] some are predominantly aggregative or ‘integrative’, where findings from individual primary qualitative studies are summarised to address specific questions, e.g., meta-summary [11]; others are mainly ‘configurative’ [12] or interpretive with the aim of developing theory or models from conceptual literature, e.g., grounded theory [13, 14] and meta-ethnography [15]. Meta-ethnography is the most frequently used QES approach in health-related research [8–10] accounting for over half of the peer-reviewed QES journal papers published between 1980 and 2010 [9]. It is an inductive, interpretive synthesis approach published in 1988 by Noblit and Hare [15], who are ethnographers in education research. They designed the approach to address the inability of an aggregative synthesis of five ethnographic studies to explain the failure of racial desegregation in schools. Although originally intended to synthesise ethnographic studies, Noblit and Hare stated that the approach could be used to synthesise interpretive qualitative studies and it has since been used to synthesise qualitative studies from a range of philosophical standpoints that have used a range of data collection techniques [5, 16].

Noblit and Hare [15] described three ways in which studies can be synthesised depending on how their findings relate to one another: a ‘reciprocal synthesis’ when concepts in one study can incorporate those of another, a ‘refutational synthesis’ when the concepts in different studies contradict one another, and a ‘line of argument synthesis’ when the studies identify different aspects of the topic under study that can be drawn together in a new interpretation. They described seven phases in their meta-ethnography approach, which can overlap and be carried out simultaneously: (1) ‘getting started’ - deciding the focus of the synthesis; (2) ‘deciding what is relevant to the initial interest’ - selecting studies to synthesise; (3) ‘reading the studies’ repeatedly and noting of metaphors, concepts, themes; (4) ‘determining how studies are related’ by juxtaposing concepts/metaphors from studies to see how they relate to each other; (5) ‘translating the studies into one another’ by comparing concepts/metaphors between and within accounts; (6) ‘synthesising translations’ by seeing if there are common types of translations or if some translations or concepts can encompass those from other studies; and (7) ‘expressing the synthesis’ - tailoring its communication to the audience. We give a more detailed description of phases 5 and 6 below and of all seven phases in Table 1.

**Table 1:** **The seven phases of Noblit and Hare’s meta-ethnography approach**

Phase	Noblit and Hare’s description
Phase 1: Getting started	‘Identifying an intellectual interest that qualitative research might inform’ ([15], p.26). The focus of the synthesis may be revised through reading interpretive qualitative studies.
Phase 2: Deciding what is relevant to the initial interest	Study selection should be ‘driven by some substantive interest derived from comparison of any given set of studies’ ([15], p.28). Searches for studies need not be exhaustive: ‘unless there is a substantive reason for an exhaustive search, generalizing from all studies of a particular setting yields trite conclusions’ ([15], p.28).
Phase 3: Reading the studies	The repeated reading of studies and noting of metaphors with close attention to details in the studies and what they tell you about your area of interest ([15], p.28).
Phase 4: Determining how the studies are related	Noblit and Hare recommended that reviewers create ‘a list of key metaphors, phrases, ideas and/or concepts (and their relations) used in each account, and [to] juxtapose them’ ([15], p.28) in order to make an initial assumption about how the studies relate to one another. This informs the type of synthesis that will be carried out – a reciprocal or refutational translation or line of argument synthesis.
Phase 5: Translating the studies into one another	The metaphors and/or concepts in each account and their interactions are compared or ‘translated’ within and across accounts while retaining the structure of relationships between central metaphors/concepts within accounts. The translations taken together are ‘one level of meta-ethnographic synthesis’ ([15], p.28). These are systematic comparisons and reciprocal translation is key to a meta-ethnography.
Phase 6: Synthesising translations	If there are many translations from phase 5 these can be compared with one another to see if there are common types of translations or if some translations or concepts can encompass those from other studies. ‘In these cases, a second level of synthesis is possible, analyzing types of competing interpretations and translating them into each other’ ([15], p.28) to reach new interpretations/conceptual understandings.
Phase 7: Expressing the synthesis	Tailoring the communication of the synthesis to the intended audience’s culture and language so that it is intelligible and meaningful to them - ‘the written synthesis is only one possible form’ ([15], p.29).

In contrast to other QES approaches, the aim of a meta-ethnography is to produce new interpretations of the primary study author’s^a^ interpretations (e.g., themes, concepts or metaphors) of the research participants’ experiences in published primary qualitative studies [6, 15, 17]. Many other QES approaches only attempt to report on or aggregate identified themes/concepts. Also unique to meta-ethnography is the systematic analysis process designed to preserve the context and meanings of the primary studies; this is underpinned by Turner’s [18] theory of social explanation which says ‘all explanation is essentially comparative and takes the form of translation’ ([15]: p. 25). Translation is the process through which data are synthesised - it involves continuously comparing the meaning of the concepts from all the primary studies to reach a full understanding of the issues [19], as such it is similar to the constant comparative approach used in primary qualitative analysis [14]. Noblit and Hare describe this process as: ‘one case [study] is like another except…’ ([15]: p. 28). Translation is idiomatic rather than literal and is intended to ‘preserve the structure of relationships between concepts within any given study’ ([17]: p. 210). However, Noblit and Hare did not describe the synthesis process (phase 6) in detail, seeing it as comparable to how qualitative research is typically conducted [19]. They described it as ‘making a whole into something more than the parts alone imply’ ([15]: p. 28), i.e. going beyond the findings of any individual study.

Noblit and Hare’s [15] seminal book gave no guidance on how to sample or appraise study reports for inclusion in a meta-ethnography (although they did not advocate formal appraisal of studies prior to synthesis) and the analytic process of synthesising was not clearly defined [20]. Since they published their book in 1988 - which to date has not been revised - there have been methodological advances, undertaken by researchers other than Noblit and Hare, in literature searching, identification (facilitated by technological innovation in cataloguing and search methods) and reporting for *quantitative* reviews of health-related research. Robust methods of study identification [21] and selection now exist [22]. The new methodological developments can apply to evidence synthesis in general and the conduct of meta-ethnography. Furthermore, guidance on selecting the most suitable QES approach to address a particular research aim [23, 24] and whether and how to assess the quality of studies for inclusion in meta-ethnographies has recently been developed [22]. Increasingly, QES experts agree that meta-ethnography is best suited to addressing a conceptual question through synthesis of a limited number of conceptually-rich studies; to synthesise a very large number of studies a thematic synthesis is often considered more appropriate [3, 19], although this view is not shared by all experts [25].

Seminal health-related meta-ethnography publications (worked examples by Britten et al. [17], Atkins et al. [20], Malpass et al. [6], Campbell et al. [16] and a methodological report by Campbell et al. [19]) have been published since the early 2000s. These attended to methods of publication identification, selection [16, 17, 19, 20] and quality appraisal [6, 20] for meta-ethnography, and further developed and clarified the meta-ethnography analytic synthesis methods [6, 16, 17, 19, 20]. For instance, Atkins et al. [20] refined the analytical methods to synthesise over 40 papers as compared to Noblit and Hare’s synthesis of five papers. More recently in 2011 Campbell et al. [19] in their ‘Evaluating meta-ethnography’ health technology assessment (HTA) report gave a detailed account of how they operationalised and conducted each of Noblit and Hare’s seven phases in two meta-ethnographies carried out as part of their evaluation of the meta-ethnography approach for synthesising health-related qualitative studies. In this report they drew together substantive methodological research from several of their earlier published meta-ethnographies [5, 16, 17]. They gave detailed worked examples of their analysis and synthesis from two separate meta-ethnographies of 38 and 25 primary studies respectively, including explicit descriptions of the translation (phase 5) and synthesis of translation (phase 6) processes. We consider the report to represent the most comprehensive methodological research on meta-ethnography (in health research) since the publication of Noblit and Hare’s [15] book. There are no examples of refutational syntheses of which we are aware, aside from Noblit and Hare’s example [15], although in some meta-ethnographies [16] reviewers^b^ have identified and excluded a paper(s) which could not be synthesised with the other papers or identified multiple lines of argument that were difficult to synthesise into a single line of argument [20].

Despite these advances in the meta-ethnography approach for health research, reviews by Dixon-Woods, Booth and Sutton [10] and Hannes and Macaitis [8] of health-related qualitative syntheses of *all* types published between 1988–2004 and 2005–2008 respectively suggested some problems with the quality of reporting in meta-ethnographies. Hannes and Macaitis commented that 14 of the 25 meta-ethnographies they identified ‘failed [..] to comply with the methodology in conducting or presenting their synthesis’ ([8]: p. 433). Dixon-Woods et al. [10] had noted similar problems. However, these reviews only touched on reporting problems – they did not focus specifically on the meta-ethnography approach, nor did they carry out an in-depth, detailed evaluation of the application and reporting of meta-ethnography.

It is recognised that poor conduct and reporting are barriers to judging the rigour, trustworthiness and credibility of the findings of any qualitative study and meta-ethnography is no exception. A lack of trust in the conduct and findings may potentially reduce the likelihood that such syntheses will be included in the range of evidence that influences future health care research, policy and practice. Reporting guidelines for QES in the form of the ‘Enhancing transparency in reporting the synthesis of qualitative research’ (ENTREQ) statement [26] have recently become available. These are generic so do not provide detailed specific guidance for individual approaches for synthesising qualitative research, such as meta-ethnography with its distinct analysis and synthesis processes. Therefore, it seems unlikely that use of ENTREQ will increase reporting quality of published meta-ethnographies, particularly of the analysis and synthesis process and output.

We do not know in detail the problems with the application and reporting of meta-ethnography methods and analysis, nor whether reporting has improved since earlier reviews [8, 10]. Given that the potential of high quality (well-conducted, well-reported) meta-ethnographies to inform research, policy and practice may be jeopardised by reporting that lacks transparency, our aim was to investigate in-depth the application and reporting of methods in recent health-related meta-ethnography journal papers published since Campbell et al’s seminal report from 2011 [19], with a specific focus on how reviewers conducted and reported the analysis and synthesis.

## Methods

We carried out a methodological systematic review of health-related meta-ethnography journal papers published from January 2012 to December 2013 (a two-year period) using content analysis [27] to establish the application and reporting of methods and findings.

### Data sources and search strategy

Current author^c^ EF searched six electronic databases on 16 December 2013 including: MEDLINE, PsycARTICLES, PsycINFO via Health Source: Nursing/Academic Edition; Pubmed; the International Bibliography of the Social Sciences; Sociological abstracts; and also searched BioMed Central journals online. The following sensitive search terms were used as keywords to search anywhere in the manuscript: meta ethnograph* or meta-ethnograph* or metaethnograph* or Noblit. The study protocol is available on request from EF. The searches identified 51 papers after exclusion of duplicates – 43 were identified via electronic databases and eight via other sources including one via Zetoc alert, four via Google Scholar searches for relevant papers that had cited Campbell et al’s 2011 HTA report [19], one by chance, and two further papers were known to the research team. The search results are shown in the PRISMA flow chart in Additional file 1: Figure S1.

### Inclusion criteria and screening of papers

To be included in our review, a paper had to meet all of the following criteria:

-  in the title and/or abstract have described their approach as meta-ethnography or as using the approach of Noblit and Hare [15] and report a synthesis of qualitative research studies
-  on a topic with a health or health care focus including those not carried out in a health care setting
-  published between January 2012 and the search date of 16 December 2013
-  published in the English language
-  published in a peer-reviewed journal.

Two of the current authors (EF and RT) independently screened all the retrieved references by title and abstract with 100 percent initial agreement on which papers were to be included. Thirty-two papers were judged to be eligible and included in the review. The data set (included papers) supporting the results of this review is included within Appendix 1. A list of excluded papers with reasons for their exclusion is given in Additional file 2: Table S1. No ethical approval was required for this methodological systematic review which involved an analysis of published journal papers and it was not eligible for inclusion in the PROSPERO database of systematic reviews with a health-related outcome.

### Data extraction and analysis

We developed a bespoke template for data extraction and recording of our analysis. We drew on meta-ethnography publications providing rich methodological detail [6, 15, 17, 19, 20] and our experience of conducting meta-ethnographies to develop questions related to the conduct of the seven phases of Noblit and Hare’s meta-ethnography approach. We were also informed by Dixon-Woods et al’s [10] and Hannes and Macaitis’ [8] reviews of qualitative syntheses for questions relating to other aspects of reviews (corresponding to Noblit and Hare’s phases 1 to 3), such as literature search and selection methods. We did not draw on ENTREQ [26] because it does not contain detailed guidance specific to the analysis and synthesis methods for meta-ethnography (phases 4 to 6) on which we intended to focus. In the analysis and discussion section we have explained the importance of each of the items analysed. In Additional file 3: Figure S2 all the items are listed.

EF drafted the data extraction template on a Microsoft^®^ Excel spreadsheet, applied it to one paper and then revised it. NR and RT then reviewed the template, independently applied it to three papers and further revisions were made after discussion. EF, RT and NR each extracted data from nine to eleven papers into the template. In addition, EF used NVivo 10 [28] to code each paper and its additional online files, if any, to allow us to further analyse their content easily and to refer back to them. The data extraction questions are shown in Additional file 3: Figure S2, the summarised and individual characteristics of papers in Additional file 4: Table S2 and Additional file 5: Table S3 respectively and details of each paper’s analytic reporting in Additional file 6: Table S4.

To check the reliability of our data extraction and analysis a second current author (NR, EF or RT) independently extracted data from a total of six (19%) papers that had been assessed already by another current author. This showed that we had low agreement on our judgements of whether or not the reviewers’ descriptions of the analysis process were clear in the included papers, especially Noblit and Hare’s phases 5 and 6, the translation and synthesis processes. Therefore NR and EF had two day-long face-to-face meetings within one week to discuss the assessment of papers in-depth and confirm or disconfirm judgements. As a result and to achieve consistency we revised our definition of ‘clearly described’ to mean that the reviewers of included papers had described the translation and synthesis processes so that we understood unambiguously what had been done. On this basis we revised some of our previous decisions regarding whether the process of translation and synthesis had been clearly described. The rationale for the final agreement reached by EF and NR was presented to RT, JN, MM and RJ who found it acceptable. All current authors had access to the papers included in the review, had opportunities to challenge or agree with NR and EF during several conference calls and email discussions, and were involved in data interpretation.

## Results and discussion

### Characteristics of papers

We identified 32 health-related meta-ethnography papers published in peer-reviewed journals in a two-year period between 2012 and 2013. This is more than Dixon-Woods et al. [10] identified (N = 19) in a sixteen-year period (1988–2004) and more than Hannes and Macaitis [8] (N = 25) identified in a four-year period (2005–2008); the average annual publication rate appears to have more than doubled since Hannes and Macaitis’ review. This substantial rise could reflect the increasing popularity of meta-ethnography in health-related research as a qualitative synthesis approach and/or that more meta-ethnographies are being funded and subsequently accepted for publication.

The papers were published in a wide range of journals, although 13 (41%) were published in a nursing or midwifery journal, slightly higher than the proportion (32%) in Hannes and Macaitis’ [8] earlier review. This indicates the continued popularity of the approach in nursing research. A large proportion of the papers focused on a disease, clinical condition or health issue (38%) covering a wide range of issues from women’s experiences of perineal trauma (paper S22) to uptake of HIV testing (paper S20) and experiences of rheumatoid arthritis (paper S7), perhaps reflecting the utility of the meta-ethnography approach for synthesising experiential data and the tendency for individual qualitative studies to explore people’s experiences. The rest of the papers focused on health interventions or other health topics, e.g., the impact of schools on child health (paper S16), the nurse-patient relationship (paper S1), and the implementation of fall-prevention programmes (paper S4). The summarised findings of the data extraction are reported in Additional file 4: Table S2.

When searching for papers, the title and abstract are the only information available to the searchers unless the full paper is retrieved so the title and abstract should enable searchers to judge the paper’s relevance. In terms of how the reviewers in this review described their studies, the titles of only 10 papers included the term ‘meta-ethnography’ while almost the same proportion used the term ‘meta-synthesis’ and/or ‘systematic review.’ Three papers did not contain any reference to the synthesis method in the title. In the abstract or methods sections 24 papers (75%) said they had used Noblit and Hare’s [15] meta-ethnography approach, whereas in eight papers reviewers said that their analysis was ‘based on’ (papers S4, S14, S18, S27, S31), ‘guided by’ (paper S15) or ‘adapted from’ (papers S13, S16) Noblit and Hare’s approach.

Reviewers of one paper (paper S13) described their paper as a worked example of a meta-ethnography, but it was not rich in detail and lacked transparency. In two papers (S7, S17) reviewers reported the findings of an updated meta-ethnography – in one of these (paper S17) they compared two of their meta-ethnographies in which they had synthesised studies published in different time periods – whereas the others were one-off meta-ethnographies. The majority of reviewers each had conducted only one of the meta-ethnographies that were included in our review.

We assessed whether each paper was recognisable as a meta-ethnography. Our criteria were that the reported methods and findings should demonstrate an inductive, interpretive synthesis that used the principles of Noblit and Hare’s [15] seven phase analytical approach (we allowed for changes to methods for searching for and selecting studies in line with advances in this field) in order to synthesise conceptual data from primary qualitative studies^d^ with the intention of reaching a new interpretation. We specified these as key principles of a meta-ethnography:

-  the reviewers had synthesised published primary qualitative studies
-  they had used an interpretive not an aggregative approach
-  reviewers had re-interpreted and synthesised *conceptual* data (even if they also synthesised some participant quotes or other descriptive data from primary studies)
-  reviewers had conducted a process which resembled translation (phase 5) as described by Noblit and Hare [15]
-  reviewers had intended to reach a new interpretation even if it was not possible for them to achieve this.

On this basis we considered that only one-third (N = 11) of the papers in our review were clearly identifiable as meta-ethnographies. See Additional file 6: Table S4 for our assessments of each paper. Although in eight papers (S4, S13-S16, S18, S27, S31) reviewers said they had somehow modified Noblit and Hare’s approach, five of these (papers S4, S13-S16) were recognisably meta-ethnographies and in one (paper S18) there was not enough information on their methods for us to be able to determine whether it was indeed a meta-ethnography. For the purposes of this review, we developed our own criteria for judging whether a study was a meta-ethnography, however, there is need for wider debate among experts about this issue, including what characterises a meta-ethnography compared to other synthesis approaches.

We found the meta-ethnography approach being applied in diverse ways supporting Campbell et al’s statement that, despite the potential of meta-ethnography as a qualitative synthesis approach, ‘it is still evolving and cannot be regarded as a standardised approach to be applied in a routinised way’ ([19]: p. 125). Some reviewers in our review had modified the meta-ethnography approach or used it in novel ways. Meta-ethnography, like any research approach, should be open to novel use as long as the methods are transparent, but we debated at what point does an adaptation of the meta-ethnography approach become something other than a meta-ethnography? We identified two key issues here: (1) reviewers adapting (‘evolving’) the approach in response to methodological or practical challenges they encountered in ways that appeared to enhance the method and that seemed to be in keeping with Noblit and Hare’s original philosophy, e.g., by separately synthesising two or more groups of papers with a like focus before attempting to do an over-arching synthesis of all studies; (2) reviewers claiming they were doing a meta-ethnography but not following the key principles of the approach (applying it inappropriately or in ways at odds with Noblit and Hare’s philosophy), for example, they had not synthesised the interpretations (conceptual data) of the primary study authors (paper S15) or they appeared to have aggregated primary study findings rather than performed a translation and synthesis of translations (e.g. paper S12).

The existence of syntheses that some reviewers claim are meta-ethnographies but that do not follow the approach could potentially damage the reputation of meta-ethnography making it less likely that users of evidence syntheses, e.g., researchers, clinical guideline developers, and policy makers, will have confidence in meta-ethnography findings. To draw a parallel, if reviewers were to publish a non-systematic literature review but call it a systematic review, this would mislead the reader and potentially devalue the reputation of systematic reviews as a rigorous and credible method. Similarly, if reviewers were to carry out some other form of qualitative synthesis (e.g., narrative or thematic synthesis) or some other type of qualitative analysis (e.g., secondary analysis of primary data) but label it a meta-ethnography then this would cause confusion and potential damage to the reputation of the approach as coherent, distinct, rigorous and systematic.

### Citation of seminal meta-ethnography methodological texts

Citing key methodological texts is not a guarantee of high quality reporting, neither is absence of these in citation lists an indicator of poor reporting. However for methodological progress and to avoid reviewers ‘reinventing the wheel’, it could be advantageous for them to build on seminal meta-ethnography methodological texts. We examined whether the papers in our review cited six seminal texts [6, 15–17, 19, 20] which included worked examples and a methodological report published between 2002 and 2011, in addition to Noblit and Hare’s [15] book. All 32 papers in our review cited Noblit and Hare [15], only 10 papers cited Britten et al. [17], 10 cited Campbell et al’s 2003 paper [16], eight cited Atkins et al. [20], and seven cited Malpass et al. [6]. Ten papers cited none of these (papers S3, S6, S12, S16, S18, S21, S23, S27, S30, S32). Reviewers of seven papers cited Campbell et al’s 2011 report [19] (although we identified four of these through their citation of this reference). Details of seminal text citation in individual papers are given in Table 2.

**Table 2:** **Citation of seminal meta-ethnography texts in methods section**

Paper	Noblit & Hare [15]	Britten et al. [17]	Campbell et al. [16]	Atkins et al. [20]	Malpass et al. [6]	Campbell et al. [19]
S1.	✓	✓	-	-	✓	-
S2.	✓	✓	-	-	-	-
S3.	✓	-	-	-	-	-
S4.	✓	-	✓	✓	-	-
S5.	✓	-	-	-	✓	-
S6.	✓	-	-	-	-	-
S7.	✓	-	✓	-	-	✓
S8.	✓	✓	✓	-	-	-
S9.	✓	-	-	-	-	✓
S10.	✓	-	-	✓	-	-
S11.	✓	✓	✓	-	✓	✓
S12.	✓	-	-	-	-	-
S13.	✓	✓	✓	-	✓	-
S14.	✓	-	-	-	-	✓
S15.	✓	-	✓	✓	-	-
S16.	✓	-	-	-	-	-
S17.	✓	✓	✓	-	-	-
S18.	✓	-	-	-	-	-
S19.	✓	-	✓	-	-	-
S20.	✓	✓	✓	✓	✓	-
S21.	✓	-	-	-	-	-
S22	✓	-	-	-	-	✓
S23.	✓	-	-	-	-	-
S24.	✓	-	-	✓	-	✓
S25.	✓	-	-	✓	-	✓
S26.	✓	-	-	✓	-	-
S27.	✓	-	-	-	-	-
S28.	✓	✓	✓	-	✓	-
S29.	✓	✓	-	-	✓	-
S30.	✓	-	-	-	-	-
S31.	✓	✓	-	✓	-	-
S32.	✓	-	-	-	-	-

Reviewers of a number of nursing papers (S3, S11, S24, S27) quoted Walsh and Downe [29] as a methodological source, however, our detailed reading of this paper did not reveal how Walsh and Downe had advanced the meta-ethnography approach in their summary and critique of the meta-ethnography methodological literature. In eight papers (S11, S13, S18, S23, S24, S29, S30, S32) reviewers cited Sandelowski and Barroso’s ‘Handbook for synthesising qualitative research’ [30] in their methods sections, although this is not specific to meta-ethnography. Reviewers of only one paper (S25) used ENTREQ (out of the 19 papers in our review published since ENTREQ’s publication) to guide their reporting, therefore it is clear that it is not being widely used. ENTREQ was not developed using methods recommended as good practice in health research reporting guideline development, such as expert consensus techniques [31], and it is unlikely to improve reporting quality of meta-ethnographies because it provides no detailed guidance on how to report the distinct analytic synthesis process. We have discussed in turn below how reviewers of papers in our review reported each of the seven phases of a meta-ethnography.

### Phase 1. ‘Getting started’

We recorded the research aim in each paper and assessed whether a meta-ethnography approach was suitable to address it, i.e. were they asking a conceptual question that could be addressed through an interpretive qualitative approach. In three (9%) papers (S5, S20, S31) reviewers did not explicitly state an aim at all. In five papers (S7, S9, S23, S29, S32) the review aim explicitly referred to the intention to develop a conceptual model, theory or new interpretation, to which meta-ethnography is well-suited. However, in four papers (S2, S4, S22, S31) the approach did not appear to suit the explicit or implicit aim: the aim of paper S2 was more suited to a quantitative study (this paper reported both a qualitative and a separate quantitative review), and the aims of papers S4, S22 and S31 implied the reviewers wanted to summarise, describe, or aggregate findings rather than re-interpret concepts. This suggested that reviewers might have had difficulty selecting the right synthesis approach, had misunderstood the purpose of meta-ethnography, or had adapted the approach for a new purpose. The aims of 19 (59%) papers (S1, S3, S6, S8, S10, S11, S13-S19, S21, S24-S28, S30) could have been met by a number of QES approaches in addition to meta-ethnography. For example, the reviewers aimed to explore experiences or perceptions of a phenomenon but did not state that they wanted to develop a conceptual model or arrive at a fresh interpretation of data from primary studies.

### Phase 2. ‘Describing what is relevant to initial interest’

#### Literature searches, study selection and quality appraisal

It is considered good practice in systematic reviews of *quantitative* research to carry out exhaustive literature searches to avoid bias in study selection [32] and to provide a full description of search and selection processes to allow them to be replicated. There is some debate as to whether exhaustive searches are necessary for qualitative reviews – an alternative approach often seen as more suited to qualitative syntheses is purposive sampling of qualitative studies [16]. In most papers, reviewers explicitly and fully reported the methods for literature searching, selecting studies for inclusion and quality appraisal. In the majority (81%) of papers reviewers used exhaustive search techniques (see Additional file 5: Table S3 and Additional file 6: Table S4). However, there was insufficient information to allow us to determine the approach to searching in four papers (S12, S21, S27, S30) and in one paper (S31) the reviewers did no literature search but had synthesised their own previously reported studies. Most reviewers (78%) provided full details of the databases searched and two-thirds gave full details of search terms. Supplementary search strategies, such as hand searching key journals, were used in two-thirds of papers. In most cases (N = 20) the literature search was limited by date range, however, in four papers this information was not provided.

The widespread adoption of exhaustive, systematic searching in these qualitative syntheses appears to indicate acceptance of these methods that were developed for quantitative reviews. It might also reflect the existence (and adoption by some journal editors) of established methods and guidance for carrying out these tasks in quantitative systematic reviews, such as the PRISMA (Preferred Reporting Items for Systematic Reviews and Meta-Analyses) statement [33] and Cochrane Collaboration [1] and University of York Centre for Reviews and Dissemination guidance on systematic reviewing [34]. However, the conditions under which exhaustive searches might be desirable or necessary when conducting a meta-ethnography require further exploration.

In quantitative systematic reviews, assessing the methodological quality of studies is required to inform data interpretation and decisions to exclude biased studies from a review [1]. In reviews of qualitative studies quality appraisal (QA) tools are sometimes used to exclude low quality studies from syntheses or to help assess their contribution to the final synthesis [35], but their use is more controversial [36]. Noblit and Hare’s [15] position was that the adequacy of the study metaphors to account for the phenomena was an important quality consideration and that study quality would become apparent by how much it contributed to the synthesis.

Quality appraisal of qualitative studies is very time-consuming, judging quality is subjective, and poor descriptions of methods (which is a key focus of QA tools) do not necessarily equate to a poorly-conducted study [37]. Nonetheless, some form of QA of retrieved or included primary studies was used by reviewers in most papers in our review, although QA was not always used to exclude papers from the synthesis. A wide range of QA tools were used overall and these are described in Additional file 5: Table S3. The Critical Appraisal Skills Programme (CASP) tool [38] was the most common method (used in over a third of papers). This prevalence of QA might reflect its desirability in *quantitative* systematic reviews and the availability of various tools for critical quality appraisal of primary qualitative study reports, e.g., the Consolidated Criteria for Reporting Qualitative research (COREQ) [39] and the Joanna Briggs Institute Qualitative Assessment and Review Instrument (JBIQARI) [40]. The most appropriate role, if any, for QA of primary qualitative studies for inclusion in a meta-ethnography still needs further debate and investigation.

There is a growing opinion among qualitative synthesis experts that meta-ethnography requires conceptually-rich papers since descriptive papers usually have too little depth to allow an interpretive synthesis [19]. They recommend that reviewers select papers on the basis of conceptual richness (which in itself can be an indicator of quality [41]) and consider the conceptual contribution of each primary study to the synthesis [3, 19]. However, reviewers of only three papers (S1, S16, S28) judged the conceptual richness of primary studies, which was done to exclude descriptive studies. Reviewers in paper S28 identified ‘key papers’ through a ‘global judgement’ comprising ‘several unspecified factors’ to identify studies conceptually rich enough to ‘potentially make an important contribution to the synthesis’ (p. e830). In papers S1 and S16 reviewers used a scale of ‘high’, ‘medium’ and ‘low’ to rate conceptual richness, which reviewers of paper S16 described as going ‘beyond a description of the findings and interpret[ing] them to develop concepts, theories or metaphors’ (p. 4), whereas in paper S1 reviewers were asked to rate the ‘weight’ of findings in terms of giving ‘rich insight’ on the research topic (p. 762). There is currently no established method for judging conceptual richness of which we are aware, although Popay, Rogers and Williams’ [41] identified several criteria for judging the adequacy of the primary study authors’ interpretation of the subjective meaning of research participants – with subjective meaning being proposed as a ‘primary marker’ of the quality of qualitative papers. However, the best ways to define and judge richness require further exploration.

#### Number of included papers

Meta-ethnography is a labour-intensive approach: Britten and Pope [37] estimated that one of their research group’s meta-ethnographies of around 40 papers took experienced reviewers 18 months part-time to conduct. Some reviewers have suggested that there is a limit to the number of papers that can be synthesised successfully using a meta-ethnography approach. Campbell et al. [19] suggested the maximum is around 40 in order to be sufficiently familiar with and immersed in all the data; Toye et al [25] contested this and have synthesised over 70 papers aided by computer software for qualitative data analysis (paper S28). Thus, how many primary studies are too many to synthesise meaningfully is still to be agreed.

Reviewers in our review synthesised from 3 to 77 papers (reporting 3 to 60 studies) with an average of 21 and a median of 18 papers. Clearly there is a trend for large numbers of studies/papers to be synthesised; in contrast Noblit and Hare [15] synthesised only five ethnographic study reports. The challenges and methods for synthesising 77 as opposed to five papers are likely to be quite different, if only because of the huge difference in the volume of data to be analysed. This indicates that the analytic methods might need adapting from Noblit and Hare’s original approach to meet the needs of present day health care research.

The methods adopted by reviewers in this review for translating and synthesising large numbers of primary studies varied. In two papers (S16, S29) reviewers grouped primary studies into smaller ‘sets’ according to their main research focus and synthesised each of these sets separately before drawing them together in an over-arching synthesis. This technique has been used by other reviewers previously [37]. In contrast, reviewers of paper S5 synthesised as a whole 34 heterogeneous primary studies with many types of dementia care workers from a wide variety of different care settings. When there is a very large number of diverse studies they can be too varied in focus and/or lacking in conceptual richness to synthesise effectively meaning that the resultant synthesis might lack depth, as was the case with paper S5. Further research is needed to identify the best methods for synthesising large numbers of studies using a meta-ethnography approach.

### Phases 3–6. Reporting of the analytic and synthesis processes

Explicit descriptions of how data were analysed and synthesised is good practice in research reporting to ensure that the reader can judge the rigour of the methods and trustworthiness of findings [42]. As Toye et al [22] point out, poor reporting of methods does not necessarily mean that the study was poor quality but that ‘there was insufficient information to make a judgement about the interpretation presented’ (p. 11). Overall, the reporting of the analysis and synthesis methods and processes in papers in our review lacked clarity. In just one third of papers reviewers fully described the analytical phases described by Noblit and Hare [15] beyond giving just a label for each phase, e.g., ‘getting started’, ‘synthesising translations’.

Some of the papers we reviewed had been condensed from much longer reports (e.g., papers S28, S32) or theses (e.g., papers S6, S17, S22, S29, S31) which might have contributed to ambiguities in reporting. Furthermore, some were published in journals with relatively short word limits of 4000 or 5000 words, e.g. papers S5, S6, S8, S9, S14, S27 and S28.

We examined the terminology reviewers used to describe their methods. There is currently no agreed, standardised terminology for some of the meta-ethnography analytical and synthesis processes and this was reflected in our findings. Some reviewers, similar to those of earlier published meta-ethnographies [6, 15, 16], drew on Schutz’s [43] notion of first and second order constructs to differentiate between the research participants’ experiences and the authors’ interpretations respectively in primary studies. Some also used the term ‘third order constructs’, coined by Britten et al [17], to refer to new interpretations resulting from a synthesis. However, contrary to Schutz’s [43] definition, the reviewers in papers S4, S6 and S24 said they had developed second order constructs from primary studies. Reviewers of paper S29 called their final synthesis output (from phase 6) a ‘fourth order construct’, a new term. Others have also noted that use of the terms first, second, and third order constructs is inconsistent in published meta-ethnographies [6] and that there seems to be confusion about the distinction between first and second order constructs [10].

The terms that reviewers used to describe the analytical processes, the conceptual data to be synthesised, and the result of the synthesis varied widely, were inconsistent across papers and often confusing to the reader making it difficult for us to follow the methods used in many papers. For instance, reviewers of paper S3 referred to ‘key concepts’ in the primary studies and ‘emerging themes across studies’ but it was unclear how these were identified and differed. Reviewers of one paper (S14) used the terms ‘line of argument synthesis’ and ‘third order interpretation’ synonymously, others said they had developed a line of argument from third order constructs or reciprocal translations (papers S16, S25). Clarity of definition and/or a shared terminology would help make the methodological processes and reporting more transparent and aid the reader’s understanding of them. Establishing any shared terminology should be done through a process of expert debate and agreement to ensure its suitability and acceptability to reviewers.

#### Phase 3. ‘Reading the studies’

In just four papers (S7, S10, S19, S31) reviewers stated the order in which they had read and synthesised papers. Atkins et al [20] argued that this order could affect the final synthesis. Some experts believe that identifying a conceptually-rich ‘index paper’ as the starting point could be the best strategy [16] – this was used in papers S6 and S24. Reviewers of paper S24 did not say how they selected the index paper and in paper S6 they chose a data-rich study of ‘acceptable methodological quality’ (p. 675). An alternative approach is to synthesise studies chronologically [6, 22] – seen in papers S7, S10, S19 and S31. To our knowledge the two contrasting methods have not been systematically compared, so it is not clear how the order of reading and synthesising papers affects the output of a synthesis, or whether one method is better than another. However, it is reasonable to suppose that the concepts presented in the paper with which a reviewer starts a meta-ethnography might have a disproportionate influence on the final conceptual output of the synthesis.

In almost three-quarters of papers (N = 23) reviewers explained how they had identified concepts or metaphors from primary studies - often by closely reading each paper and noting down themes in a matrix - whereas reviewers of only nine papers stated how many themes or concepts they had identified from primary studies. This can make it difficult for the reader to follow the development of the reviewers’ new interpretation of the data in primary studies and, more importantly, to judge whether a new interpretation was indeed achieved.

#### Phase 4. ‘Determining how studies are related’

In the majority of papers (81%) reviewers stated they had carried out phase 4 of Noblit and Hare’s approach of determining how the studies they intended to synthesise were related. However, in only two-thirds (N = 21) of papers did the reviewers clearly describe *how* they had determined the relations between primary studies, usually by comparing themes/concepts across the studies to determine a reciprocal, refutational or a line of argument relation between them. In four (13%) papers (S2, S4, S9, S28) reviewers did not provide any description of how, or if, they had conducted this stage of the synthesis process.

#### Phase 5. ‘Translating the studies into one another’

No reviewers carried out a refutational synthesis, all were reciprocal or line of argument syntheses. This is perhaps unsurprising given the lack of refutational syntheses in previous published health-related meta-ethnographies. However, it could be argued that reviewers should seek out primary studies containing contradictory concepts as part of the translation process, in line with the principles of the constant comparative analytic method in which disconfirming or contradictory cases are used to test emerging understandings of the data [14].

It was difficult to discern how phase 5, the translation process had been carried out in most papers: in less than one third of papers (N = 10) (papers S1, S10, S12, S16, S17, S19, S20, S22, S25, S32) reviewers gave a clear description. Some reviewers said they had translated concepts but did not say how (papers S12, S23, S29, S30) or gave a vague description of their process, e.g., ‘developing common categories’ (paper S31, p. 5). Other reviewers appeared to have used constant comparison, i.e. comparing and contrasting the primary study authors’ concepts across primary studies to identify similarities and dissimilarities (e.g., papers S1, S5, S10, S11, S13, S16, S20, S26, S27, S28, S32). In papers S10, S16 and S20 reviewers described their process in similar terms to earlier meta-ethnographies by Pound et al. [5], Campbell et al. [19] and Britten and Pope [37] referring to comparing the main concepts from paper one with paper two, and the syntheses of these two papers with paper three, and so on. One reviewer explicitly referred to doing a thematic analysis (paper S4) and others described a process of grouping like concepts (e.g., papers S9, S17, S18) similar to a thematic analysis. In five papers (S2, S4, S5, S19, S25) reviewers referred to coding data from primary studies. We have given examples of reporting of phase 5 from papers in Additional file 7: Table S5.

In two papers (S15, S31), and possibly a third (paper S24) in which the reporting was ambiguous, reviewers did not adhere to the principles of Noblit and Hare’s translation process because they did not re-interpret concepts from primary studies: paper S31 appeared to be a secondary analysis of raw data from three previous studies carried out by the reviewers, and the reviewers of paper S15 (and possibly paper S24) had carried out an interpretive analysis of selected participant quotes from primary studies. Reviewers in paper S13 identified repeated ‘emotion words’ in primary studies and then used these, rather than translated concepts, as the basis of their analysis – this process could be considered identification of ‘metaphors’ as Noblit and Hare describe them, although the reviewers did not specifically state this. A number of papers (e.g., S5, S8, S9, S10, S11, S13, S17, S25, S32) drew on both participant quotes and primary study authors’ concepts rather than just the latter in the translation process – this differs from how Noblit and Hare described translation but seems to be fairly common practice [44]. Another paper (S28) was unique in that the reviewers had modified phase 5 by creating and using as data their research team’s joint descriptions of the concepts in primary studies rather than the concepts as described by the authors of the primary studies (they defend and further explain their novel approach in a recent paper [25]). It is not clear what might be gained or lost through such an approach to translation and whether or not it can actually be considered ‘translation’ as Noblit and Hare characterised it. The pros and cons of different methods of translation require exploration with further methodological research.

#### Phase 6. ‘Synthesizing translations’

Reporting the process used to synthesise conceptual data in a transparent way can help readers judge the rigour of the analysis. In only one paper (S10) did the reviewers unambiguously describe how they synthesised translations (phase 6). This meant that for most papers we could not easily judge the trustworthiness and credibility of their findings because it was uncertain how they were derived. There was, however, wide variation across the 32 papers in the depth of detail about the analytic process, for example, in a further eight papers (S3, S9, S13, S16, S17, S25, S29, S32) reviewers provided fairly detailed descriptions of phase 6 but fell short of an unambiguous description.

Most reviewers stated that they had conducted a synthesis of translations but not how, for instance, a number of them (papers S1, S5, S20, S24, S29) said they had developed third order constructs from second order constructs, but not how they had done this. In at least two papers (S6, S13) the final synthesis appeared to be the output of phase 5, although the reviewers did not directly state this. We give illustrative examples of transparent and opaque reporting of phases 5 and 6 in Additional file 7: Table S5. Phases 5 and 6 of the meta-ethnography approach, although labelled as individual phases, are in reality interlinked and iterative as described by Noblit and Hare [15] themselves, e.g., synthesis starts during translation. Furthermore, Noblit and Hare were not explicit about how to conduct phase 6. This could partly explain why reviewers struggled to give a distinct description of phase 6. Reviewers could benefit from a comprehensive guide about how to conduct a meta-ethnography that pulls together and extends learning from seminal publications.

An example of transparent reporting of one aspect of the synthesis process was paper S14 in which the reviewers gave tables which listed all the concepts developed by primary study authors showing which studies they came from and how these had informed new interpretations. This helped us to follow the development of the reviewers’ interpretation. However, in more than half of papers reviewers did not clearly identify which of the primary studies contributed to the professed new interpretation making it difficult to judge whether or not reviewers had simply aggregated primary studies. This lack of clarity and comprehensiveness in reporting of the analysis confirmed the indications from Dixon-Woods et al. [10] and Hannes and Macaitis [8] for earlier health-related meta-ethnographies.

Most reviewers claimed that they had developed and presented a new interpretation of the conceptual data from the primary studies they had synthesised, but in seven papers (S2, S7, S15, S23, S24, S27, S30) they did not actually present recognisable third order constructs, a line of argument or a conceptual model/theory. Only in paper S7 did the reviewers state that this was because no new conceptual development had taken place following early conceptually-rich primary studies. In over a third of papers (N = 12) it was unclear from the information given if the reviewers had actually achieved a new interpretation because the analysis process was not comprehensively described and/or it was unclear whether they had simply aggregated the authors’ concepts from the primary studies (papers S3-S5, S8, S11-S13, S18, S21, S26, S28, S31). A new interpretation may not be achieved in a meta-ethnography, for instance, in a field that reaches saturation early on with conceptually-rich primary studies [19], when primary studies are conceptually ‘thin’ (descriptive), when there are very few relevant studies, or when the focus of the synthesis constrains conceptual innovation [45], however, we would expect the reviewers to clearly state if no new interpretation was possible, as the reviewers of paper S7 did.

#### Context of interpretation in phases 5 and 6

Qualitative interpretation is usually richer and more rigorous when two or more researchers are involved [45] since each researcher brings a different perspective. Indeed Lee et al. [44] found a collaborative approach to conducting meta-ethnographies beneficial for identifying the range of possible analytic interpretations. In this review we found that the most common number of reviewers involved in the analysis and synthesis was two or three. However, in over a quarter of papers (N = 9) reviewers did not state how many of them were involved; two of these papers were by a single person probably indicating a single reviewer (papers S12, S21). In a further five papers (S1, S5, S14, S19, S22) analysis was carried out by a single reviewer, although in all of these cases others acted in a ‘review’ or ‘consultation’ capacity.

We also recorded data on reviewers’ conflicts of interest and the source of research funding because these might have influenced data interpretation. In only one paper (S25) competing interests were listed – these were the funding sources of two of the reviewers. In two-thirds of papers (N = 21) the reviewers stated they had no conflict of interest or competing interests but this information was not given in 11 papers. The United Kingdom’s (UK) National Institute for Health Research (NIHR) wholly or partly funded six (19%) of the reviews and the UK National Health Service wholly or partly funded four (13%) - together they funded almost a third of all the meta-ethnographies. Five papers reported meta-ethnographies funded by universities, two were funded by the Canadian Institutes of Health Research, two were unfunded, and five declared other funding sources. In nine (28%) papers reviewers did not state their source of funding. Funding details for each paper are listed in Additional file 5: Table S3.

#### Phase 7 – ‘Expressing the synthesis’

Noblit and Hare [15] emphasised the importance of tailoring meta-ethnography reports to their intended audience, so we examined how reviewers presented their findings. The style adopted by some was descriptive rather than conceptual (e.g., papers S4, S5, S20, S26) and/or read like a narrative synthesis, in which they had aggregated findings from primary studies, or a thematic synthesis (e.g., papers S2, S5, S12, S21, S26). Currently there is no consensus over which types of data from primary studies, e.g., quotes from participants or authors, are best used in support of meta-ethnography findings. The majority of papers (N = 26) included selected participant quotes from primary studies in their findings sections but in one paper (S9) it was not clear if the quotes given were those from primary study participants or authors. Only the reviewers of paper S5 stated they had presented quotes from the primary study authors. In half of the papers reviewers expressed their findings only in written form (including in tables). The other half used a combination of textual description and diagrams or other visual depictions of models to express their synthesis, which can be a very effective way to communicate them.

In terms of the best examples of reporting of the analytic and synthesis phases, the only paper in which reviewers unambiguously reported both phase 5 (translation of studies) and 6 (synthesising translations) was paper S10. In papers S25 and S32 reviewers unambiguously described phase 5 and gave a quite detailed description of phase 6. Overall reporting of phases 3 to 5 was clear and detailed in papers S1 and S19. Reviewers of paper S28 gave a particularly comprehensive and transparent account of literature searches, study selection and quality appraisal (phases 3 and 4).

### Raising reporting standards

Transparent reporting that clearly conveys the methodology, analysis and findings of a synthesis can help readers to judge the rigour, credibility and trustworthiness of the findings; both poor and rigorous research conduct become more apparent with good reporting. There is evidence that reporting guidelines, for example, the CONSORT statement for randomised controlled trials [46], can raise reporting standards [47]. Research reporting guidelines are specifically intended to standardise and improve the quality of research *reporting* rather than research *conduct*, although they might also improve conduct by informing research design. We believe that such guidelines can and should allow for flexibility in research conduct to avoid stifling innovative use and evolution of the methodology.

Recently, method-specific reporting guidelines, known as ‘RAMESES’ publication standards, have been developed for two other unique qualitative synthesis approaches - realist and meta-narrative reviews [48, 49] - demonstrating that it is possible to create reporting standards for complex qualitative synthesis approaches. The RAMESES guidelines [48, 49], which were developed using a robust methodology involving systematic reviews and expert consensus techniques, contain a list of items to be reported accompanied by a detailed document giving examples of good reporting and the rationale for including each item in published reviews. We believe that evidence-based meta-ethnography reporting guidelines, developed along similar lines as RAMESES, which articulate the methodological standards and depth of reporting required could improve meta-ethnography reporting.

There remain many questions for further methodological research and for debate amongst qualitative synthesis and meta-ethnography experts that we have started to explore in our review including: what are the defining characteristics (key principles) of a meta-ethnography compared to other synthesis approaches? At what point is an adapted meta-ethnography no longer a meta-ethnography? When are study search and selection methods derived from quantitative systematic reviews necessary or useful in qualitative reviews? What is the most appropriate role, if any, for quality appraisal of primary studies in meta-ethnography? How can we best judge conceptual richness of primary qualitative study findings? What terms should reviewers use to describe their meta-ethnography analytic processes, outputs and data? How many primary studies are too many to meaningfully synthesise and what are the best methods for synthesising large numbers of studies using a meta-ethnography approach? What difference does the order of reading and synthesising papers, e.g., in chronological order versus starting with a data-rich ‘key paper’, make to the output of the synthesis? How do different approaches to the translation process affect the synthesis output? Is refutational synthesis (or searching for disconfirming cases) a necessary aspect of a good quality meta-ethnography and its reporting? While we have offered our views and presented current thinking on these questions in the analysis and discussion section, we consider that they can only be addressed satisfactorily through further methodological work and/or debate amongst the wider international community of qualitative synthesis and meta-ethnography experts.

### Strengths and limitations of our review

In our review we used a rigorous analytic approach: data extraction involved three qualitative researchers from different disciplinary backgrounds (psychology, nursing and health visiting, midwifery), two experienced (EF and NR) and one more novice (RT). Our wider multi-disciplinary team involved in analysis and interpretation included a medical sociologist (MM), nurses (NR, JN, RJ) and a systematic reviewer (RJ). The team has expertise in conducting systematic reviews, qualitative syntheses and meta-ethnographies. Our views and judgements about reporting clarity are subjective and based on our interpretations of the papers included in this review, therefore we aimed to be as transparent as possible about our processes and criteria. Our criteria for clarity were demanding specifying that the processes should be unambiguous.

We restricted our searches to the most recent two years of published meta-ethnographies because we wanted to explore the latest trends post publication of Campbell et al’s 2011 seminal HTA report ‘Evaluating Meta-ethnography’ [19]. Screening by title and abstract only is established practice in systematic reviewing, however this means that we are likely to have excluded some relevant papers. In addition, our search terms and searching methods may have missed some relevant papers, e.g., if reviewers had only specified their synthesis approach as meta-ethnography in the full text, had published a paper only in a major funder’s library, or if papers were not published in English. We did not explore the full range of possible influences on the reviewers’ data interpretation, assess the quality of the reviewers’ findings, nor the impact of multi-disciplinary team analysis on quality of findings. These areas could be explored in future research. Nonetheless, we believe that our review has revealed important insights into current use of this interpretive qualitative synthesis approach and that it is the first in-depth methodological systematic review of meta-ethnography conduct and reporting.

## Conclusions

Meta-ethnography is an evolving approach which is being applied in diverse ways. There is need for expert debate on what defines a meta-ethnography and differentiates it from other QES approaches. Current reporting standards of meta-ethnography journal papers are poor overall. Inadequate reporting is a significant barrier to their utility: it means that end users, such as researchers, clinicians and clinical guideline developers, cannot assess methodological rigour and the credibility and trustworthiness of the findings of a meta-ethnography. To realise the high potential value of meta-ethnography to contribute to the health care evidence base requires transparent reporting that clearly conveys the methodology, analysis and findings. We do not believe that this will be achieved through generic qualitative synthesis guidelines because analytic synthesis approaches differ greatly. We propose that meta-ethnography’s unique, complex analysis methods would benefit from comprehensive guidance on its conduct and a method-specific reporting guideline. Furthermore, we suggest that meta-ethnography reporting guidelines should be based on expert consensus on what constitutes a well-conducted meta-ethnography and what level of reporting would allow us to judge this. Our review provides evidence on which to start discussions.

## Endnotes

^a^We have used the term ‘primary study authors’ or ‘authors of primary studies’ to refer to the authors of published primary qualitative studies.

^b^We have used the term ‘reviewers’ for the authors of meta-ethnography papers included in our review and to refer to people who carry out and publish meta-ethnographies.

^c^In the main manuscript we have referred to ourselves as the ‘current authors’.

^d^We refer to published qualitative research accounts that reviewers synthesised as ‘primary studies’ or ‘primary qualitative studies’.

## Appendix 1

### Papers included in the review

S1. Bridges J, Nicholson C, Maben J, Pope C, Flatley M, Wilkinson C, Meyer J, Tziggili M: **Capacity for care: meta‒ethnography of acute care nurses’ experiences of the nurse‒patient relationship.** Journal of advanced nursing 2013, **69**(4), 760–772.

S2. Brohan E, Henderson H, Wheat K, Malcolm E, Clement S, Barley EA, Slade M, Thornicroft G: **Systematic review of beliefs, behaviours and influencing factors associated with disclosure of a mental health problem in the workplace**, BMC Psychiatry 2012, **12**:11.

S3. Cairns V, Murray C: **How do the features of mindfulness-based cognitive therapy contribute to positive therapeutic change? A meta-synthesis of qualitative studies**, Behavioural and Cognitive Psychotherapy 2013, **11**:11–18.

S4. Child S, Goodwin V, Garside R, Jones-Hughes T, Boddy K, Stein K: **Factors influencing the implementation of fall-prevention programmes: a systematic review and synthesis of qualitative studies**, Implementation Science 2012, 7–91. doi:10.1186/1748-5908-7-91.

S5. Cook C, Fay S, Rockwood K: **A meta-ethnography of paid dementia care workers’ perspectives on their jobs**, Canadian Geriatrics Journal 2012, **15** (4):127–136.

S6. Dahl, B, Fylkesnes, AM, Sorlie, V, Malterud, K: **Lesbian women’s experiences with healthcare providers in the birthing context,** Midwifery 2013, **29**:674–681.

S7. Daker-White G, Donovan J, Campbell R: **Redefined by illness: meta-ethnography of qualitative studies on the experience of rheumatoid arthritis.** Disability & Rehabilitation 2014, **36**(13):1061–1071 (doi:10.3109/09638288.2013.829531). First published online 2013.

S8. de Sousa Pinto JM, Martín-Nogueras AM, Morano MT, Macêdo TE, Arenillas JI, Troosters T: **Chronic obstructive pulmonary disease patients’ experience with pulmonary rehabilitation: a systematic review of qualitative research,** Chronic Respiratory Disease 2013, **10**(3):141–57.

S9. Embuldeniya G, Veinot P, Bell E, Bell M, Nyhof-Young J, Sale JE, Britten N: **The experience and impact of chronic disease peer support interventions: A qualitative synthesis.** Patient education and counselling 2013, **92**(1):3–12.

S10. Franzel B, Schwiegershausen M, Heusser P, Berger B: **Individualised medicine from the perspectives of patients using complementary therapies: a meta-ethnography approach,** Complementary and Alternative Medicine 2013, **13**:124. doi:10.1186/1472-6882-13-124.

S11. Furuta M, Sandall J, Bick D: **Women’s perceptions and experiences of severe maternal morbidity - a synthesis of qualitative studies using a meta-ethnographic approach**, Midwifery 2013, pii: S0266-6138(13)00275-1. doi:10.1016/j.midw.2013.09.001. [Epub ahead of print]

S12. Galuska LA: **Cultivating Nursing Leadership for Our Envisioned Future.** Advances in Nursing Science 2012, **35**(4):333–345.

S13. Grose J, Frost J, Richardson J, Skirton H: **Using meta-ethnography to understand the emotional impact of caring for people with increasing cognitive impairment.** Nursing & health sciences 2013, **15**(1):113–123.

S14. Higginbottom G, Hadziabdic E, Yohani S, Paton P: **Immigrant women’s experience of maternity services in Canada: A meta-ethnography**. Midwifery 2014, **30**(5): 544–559.

S15. Hubbard G, McLachlan K, Forbat L, Munday D: **Recognition by family members that relatives with neurodegenerative disease are likely to die within a year: A meta-ethnography.** Palliative Medicine 2012, **26**(2):108–122.

S16. Jamal F, Fletcher A, Harden A, Wells H, Thomas J, Bonell C: **The school environment and student health: a systematic review and meta-ethnography of qualitative research**. BMC public health 2013, **13**(1):798.

S17. Lang H, France E, Williams B, Humphris G, Wells M: **The psychological experience of living with head and neck cancer: a systematic review and meta‒synthesis**. Psycho‒Oncology 2013, **22**(12):2648–2663.

S18. Lundgren I, Begley C, Gross MM, Bondas T: **‘Groping through the fog’: a metasynthesis of women’s experiences on VBAC (Vaginal birth after Caesarean section).** BMC pregnancy and childbirth 2012, **12**(1):85.

S19. Monforte-Royo C, Villavicencio-Chávez C, Tomás-Sábado J, Mahtani-Chugani V, Balaguer A: **What lies behind the wish to hasten death? A systematic review and meta-ethnography from the perspective of patients.** PloS one 2012, **7**(5):e37117.

S20. Musheke M, Ntalasha H, Gari S, Mckenzie O, Bond V, Martin-Hilber A, Merten S: **A systematic review of qualitative findings on factors enabling and deterring uptake of HIV testing in Sub-Saharan Africa.** BMC public health, **13**(1):220.

S21. Nelson AM: **A Meta-Synthesis Related to Infant Feeding Decision Making.** MCN: The American Journal of Maternal/Child Nursing 2012, **37**(4):247–252.

S22. Priddis H, Dahlen H, Schmied, V: **Women’s experiences following severe perineal trauma: a meta‒ethnographic synthesis.** Journal of advanced nursing 2013. **69**(4):748–759.

S23. Rudolfsson G, Berggern I: **Nursing students’ perceptions on the patient and the impact of the nursing culture: a meta-synthesis**. J Ng Management 2012, **20** 771–781.

S24. Schmied V, Olley H, Burns E., Duffy M, Dennis CL, Dahlen H: **Contradictions and conflict: A meta-ethnographic study of migrant women’s experiences of breastfeeding in a new country**. BMC Pregnancy and child health, 2012, **12**(1):c163.

S25. Sinnott C, Mc Hugh S, Browne J, Bradley C: **GPs’ perspectives on the management of patients with multimorbidity: systematic review and synthesis of qualitative research.** BMJ open 2013, **3**(9):e003610.

S26. Smith T, Purdy R, Lister S, Salter C, Fleetcroft R, Conaghan P: **Attitudes of people with osteoarthritis towards their conservative management: systematic review and meta-ethnography.** Rheumatol Int, 2013 Dec.

S27. Steen M, Downe S, Bamford N, Edozien L: **Not patient and not visitor: a meta-synthesis fathers’ encounters with pregnancy, birth and maternity care**. Midwifery 2012, **28**:422–431

S28. Toye F, Andrews J, Barker K, Seers K, Allcock N, Briggs M, Carr E: **Patients’ experiences of chronic non-malignant musculosketal pain**. BJGP 2013, e829.

S29. Tranvag O, Petersen K, Naden D: **Dignity preserving dementia care: a meta synthesis.** Nursing Ethics 2013, **20**:861.

S30. Tuthill E, McGrath J, Young S: **Commonalities and differences in infant feeding attitudes and practices in the context of HIV in Sub-Saharan africa: a meta synthesis.** AIDS care 2014, **26**(2):214–225. First published online 23 July 2013.

S31. Uebel K, Guise A, Georgey D, Colvin C, Lewin S: **Integrating HIV care into nurse led primary health care services in S. Africa: a synthesis of three linked studies**. BMC health services research 2013, **13**:171.

S32. Wells M, Williams B, Firnigl D, Lang H, Coyle J, Kroll T, MacGillivray, S: **Supporting ‘work‒related goals’ rather than ‘return to work’ after cancer? A systematic review and meta‒synthesis of 25 qualitative studies.** Psycho‒Oncology 2013, **22**(6):1208–1219.

## Authors’ information

EF (PhD in psychology, MA honours) is a lecturer in the NMAHP Research Unit, School of Health Sciences, University of Stirling. NR (PhD, MSc, BSc, Dip HV, RGN and RSCN) is a senior lecturer in the School of Health Sciences, University of Stirling who conducts and teaches QES. RT (RM) is a practising midwife and part-time final year student on the Masters in Health Research programme in the School of Health Sciences, University of Stirling. MM (PhD, MA honours) is Professor of Health Services and Mental Health Research and the deputy director of the NMAHP Research Unit, University of Stirling and Glasgow Caledonian University. RJ (PhD, MSc, RN) is a former Cochrane Collaboration systematic reviewer who conducts and teaches QES. JN (RN, MSc, DPhil) is co-chair of the Cochrane Methods Executive, lead convenor of the Cochrane Qualitative and Implementation Methods group, editor of the Journal of Advanced Nursing, and member of the National Institute for Health and Care Excellence (NICE) methodological hub consortium.

## Electronic supplementary material

Additional file 1: Figure S1: PRISMA 2009 Flow Diagram. (DOC 29 KB)

Additional file 2: Table S1: Papers excluded from the review with reasons. (DOCX 24 KB)

Additional file 3: Figure S2: Data extraction items and questions. (DOCX 19 KB)

Additional file 4: Table S2: Summary of characteristics of papers included in the review. (DOCX 24 KB)

Additional file 5: Table S3: Characteristics of individual included papers and reporting of meta-ethnography Phases 1 and 2. (DOCX 38 KB)

Additional file 6: Table S4: Reporting of meta-ethnography analytic phases 3 to 7 in included papers. (DOCX 29 KB)

Additional file 7: Table S5: Illustrative examples of analysis and synthesis process reporting in papers included in review. (DOCX 30 KB)

## Abbreviations

CASP: The Critical Appraisal Skills Programme
CONSORT: Consolidated Standards Of Reporting Trials
ENTREQ: Enhancing transparency in reporting the synthesis of qualitative research
EPPI-centre: Evidence for Policy and Practice Information and Co-ordinating Centre HTA - health technology assessment
JBI: Joanna Briggs Institute
JBIQARI: Joanna Briggs Institute Qualitative Assessment and Review Instrument
MESH: Medical subject headings
NIHR: National Institute for Health Research
PRISMA: Preferred Reporting Items for Systematic Reviews and Meta-Analyses
QA: Quality appraisal
QES: Qualitative evidence synthesis
UK: United Kingdom.
