# Supplementary material for: A methodological systematic review of what’s wrong with meta-ethnography reporting
Source: BMC Med Res Methodol. 2014 Nov 19;14:119. doi: 10.1186/1471-2288-14-119 (PMC4277825; doi:10.1186/1471-2288-14-119)
Supplement: Supplementary file 1 — Additional file 1: Figure S1: PRISMA 2009 Flow Diagram. (DOC 29 KB) [file 12874_2014_1138_MOESM1_ESM.doc]

**Screening**

**Included**

**Eligibility**

**Identification**

Records identified through database searching
(n = 52)

Additional records identified through other sources
(n = 8)

Records after duplicates removed
(n = 51)

Records screened
(n = 51 )

Records excluded
(n = 18)

Full-text articles assessed for eligibility
(n = 33)

Full-text articles excluded, reason – not described as a meta-ethnography
(n = 1)

Studies included in review
(n = 32)
