# Supplementary material for: A methodological systematic review of what’s wrong with meta-ethnography reporting
Source: BMC Med Res Methodol. 2014 Nov 19;14:119. doi: 10.1186/1471-2288-14-119 (PMC4277825; doi:10.1186/1471-2288-14-119)
Supplement: Supplementary file 2 — Additional file 2: Table S1: Papers excluded from the review with reasons. (DOCX 24 KB) [file 12874_2014_1138_MOESM2_ESM.docx]

**Table S1. Papers excluded from the review with reasons.**

| **Paper** | **Reasons for exclusion** |
| --- | --- |
| Barley E, Haddad M, Simmonds R, Fortune Z, Walters P, Murray J, et al. The UPBEAT depression and coronary heart disease programme: using the UK medical research council framework to design a nurse-led complex intervention for use in primary care. BMC Family Practice 2012;13(1):119. | Not described as meta-ethnography in title or abstract (synthesis of both qualitative and quantitative papers) |
| Bayhakki, Hatthakit U. Lived Experiences of Patients On Hemodialysis: A Meta-Synthesis. Nephrology Nursing Journal 2012 Jul;39(4):295-305. | Not described as meta-ethnography in title or abstract |
| Brown K, Worrall LE, Davidson B, Howe T. Living successfully with aphasia: A qualitative meta-analysis of the perspectives of individuals with aphasia, family members, and speech-language pathologists. International Journal of Speech-Language Pathology 2012 Apr;14(2):141-55. | Not described as meta-ethnography in title or abstract (qualitative meta-analysis) |
| Clark AM, King-Shier KM, Spaling MA, Duncan AS, Stone JA, Jaglal SB, et al. Factors influencing participation in cardiac rehabilitation programmes after referral and initial attendance: qualitative systematic review and meta-synthesis. Clinical Rehabilitation 2013 Oct;27(10):948-59. | Not described as meta-ethnography in title or abstract |
| Cohen K, Collens P. The impact of trauma work on trauma workers: A metasynthesis on vicarious trauma and vicarious posttraumatic growth. Psychological Trauma: Theory, Research, Practice, and Policy 2013 Nov;5(6):570-80. | Not described as meta-ethnography in title or abstract |
| Corvol A, Moutel Gg, Gagnon D, Nugue M, Saint-Jean O, Somme D. Ethical issues in the introduction of case management for elderly people. Nursing Ethics 2013 Feb;20(1):83-95. | Not described as meta-ethnography in title or abstract (primary qualitative study) |
| Eijzenga, W., Hahn, D. E., Aaronson, N. K., Kluijt, I., & Bleiker, E. M. (2013). Specific psychosocial issues of individuals undergoing genetic counseling for cancer–A literature review. *Journal of genetic counseling*, 1-14. | Could not locate full paper within timeframe of review - (note: paper identified through Google Scholar, full paper was published April 2014) |
| Franzel B, Heusser P, Lauche R, Schwiegershausen M, Berger B. P05.43. Meta-ethnography: the perspective of patients choosing alternative and complementary medicine regarding individualized medicine and integrative care. BMC Complementary and Alternative Medicine 2012;12(Suppl 1):403. | Not a peer-reviewed journal paper (conference presentation) |
| Franzel B, Schwiegershausen M, Heusser P, Berger B. How to locate and appraise qualitative research in complementary and alternative medicine. BMC Complementary and Alternative Medicine 2013;13(1):125. | Not described as meta-ethnography in title or abstract (methodological) |
| Gallacher K, Jani B, Morrison D, Macdonald S, Blane D, Erwin P, et al. Qualitative systematic reviews of treatment burden in stroke, heart failure and diabetes - Methodological challenges and solutions. BMC Medical Research Methodology 2013;13(1):10. | Not described as meta-ethnography in title or abstract (methodological) |
| Gough D, Thomas J, Oliver S. Clarifying differences between review designs and methods. Systematic Reviews 2012;1(1):28. | Not described as meta-ethnography in title or abstract (methodological) |
| Hannes K, Booth A, Harris J, Noyes J. Celebrating methodological challenges and changes: reflecting on the emergence and importance of the role of qualitative evidence in Cochrane reviews. Systematic Reviews 2013;2(1):84. | Not described as meta-ethnography in title or abstract (methodological) |
| Hughes S, Noblit G, Cleveland D. Derrick Bell's post-Brown moves toward critical race theory. Race ethnicity and education 2013 Sep;16(4):442-69. | Not described as meta-ethnography in title or abstract |
| Kangasniemi M, Halkoaho A, Länsimies-Antikainen H, Pietilä AM. Duties of the patient: A tentative model based on metasynthesis. Nursing Ethics 2012 Jan;19(1):58-67. | Not described as meta-ethnography in title or abstract |
| Kastner M, Makarski J, Hayden L, Durocher L, Chatterjee A, Brouwers M, et al. Making sense of complex data: a mapping process for analyzing findings of a realist review on guideline implementability. BMC Medical Research Methodology 2013;13(1):112. | Not described as meta-ethnography in title or abstract (methodological) |
| Kastner M, Tricco AC, Soobiah C, Lillie E, Perrier L, Horsley T, et al. What is the most appropriate knowledge synthesis method to conduct a review? Protocol for a scoping review. BMC Medical Research Methodology 2012;12(1):114. | Not described as meta-ethnography in title or abstract (protocol for a scoping review) |
| Kroos K. Eclecticism as the Foundation of Meta-theoretical, Mixed Methods and Interdisciplinary Research in Social Sciences. Integrative Psychological & Behavioral Science 2012 Mar;46(1):20-31. | Not described as meta-ethnography in title or abstract (discussion paper) |
| Levack WMM. The role of qualitative metasynthesis in evidence-based physical therapy. Physical Therapy Reviews 2012 Dec;17(6):390-7. | Not described as meta-ethnography in title or abstract (methodological) |
| Maddox R, Davey R, Cochrane T, Lovett R, van der Sterren A. Study protocol - Indigenous Australian social networks and the impact on smoking policy and programs in Australia: protocol for a mixed-method prospective study. BMC Public Health 2013;13(1):879. | Not described as meta-ethnography in title or abstract (study protocol) |
| Nagata J, Hernandez-Ramos I, Kurup A, Albrecht D, Vivas-Torrealba C, Franco-Paredes C. Social determinants of health and seasonal influenza vaccination in adults [greater than or equal to] 65 years: a systematic review of qualitative and quantitative data. BMC Public Health 2013;13(1):388. | Not described as meta-ethnography in title or abstract (thematic synthesis) |
| Newell ML, Newell TS, Looser J. A competency-based assessment of school-based consultants’ implementation of consultation. Training and Education in Professional Psychology 2013 Nov;7(4):235-45. | Not described as meta-ethnography in title or abstract (qualitative case study design) |
| Noblit GW. Culture bound: Science, teaching and research. Journal of Research in Science Teaching 2013 Feb;50(2):238-49. | Not described as meta-ethnography in title or abstract (discussion paper) |
| Reed MC, Wood V, Harrington R, Paterson J. Developing stroke rehabilitation and community services: a meta-synthesis of qualitative literature. Disability & Rehabilitation 2012 Apr;34(7):553-63. | Not described as meta-ethnography in title or abstract |
| Ring N, Jepson R, Pinnock H, Wilson C, Hoskins G, Wyke S, et al. Developing novel evidence-based interventions to promote asthma action plan use: a cross-study synthesis of evidence from randomised controlled trials and qualitative studies. Trials 2012;13(1):216. | Not described as meta-ethnography in title or abstract |
| Siabani S, Leeder S, Davidson P. Barriers and facilitators to self-care in chronic heart failure: a meta-synthesis of qualitative studies. SpringerPlus 2013;2(1):320. | Not described as meta-ethnography in title or abstract |
| Tong A, Flemming K, McInnes E, Oliver S, Craig J. Enhancing transparency in reporting the synthesis of qualitative research: ENTREQ. BMC Medical Research Methodology 2012;12(1):181. | Not described as meta-ethnography in title or abstract (reporting guidelines) |
| Toye F, Seers K, Allcock N, Briggs M, Carr E, Andrews J, et al. 'Trying to pin down jelly' - exploring intuitive processes in quality assessment for meta-ethnography. BMC Medical Research Methodology 2013;13(1):46. | Not described as meta-ethnography in title or abstract (methodological) |
| van Wesel F, Boeije H, Alisic E, Drost S. I'll be working my way back: A qualitative synthesis on the trauma experience of children. Psychological Trauma: Theory, Research, Practice, and Policy 2012 Sep;4(5):516-26. | Not described as meta-ethnography in title or abstract (thematic synthesis) |
| Welch V, Petticrew M, O'Neill J, Waters E, Armstrong R, Bhutta Z, et al. Health equity: evidence synthesis and knowledge translation methods. Systematic Reviews 2013;2(1):43. | Not described as meta-ethnography in title or abstract (methodological) |
