# Supplementary material for: A methodological systematic review of what’s wrong with meta-ethnography reporting
Source: BMC Med Res Methodol. 2014 Nov 19;14:119. doi: 10.1186/1471-2288-14-119 (PMC4277825; doi:10.1186/1471-2288-14-119)
Supplement: Supplementary file 3 — Additional file 3: Figure S2: Data extraction items and questions. (DOCX 19 KB) [file 12874_2014_1138_MOESM3_ESM.docx]

| **Description**   1. Reference (full citation) 2. Journal name 3. Journal word limit for published articles 4. Method label in title 5. Method label in abstract (phrase) 6. Method label in methods or discussion (phrase)   **Phase 1**   1. Aim (describe in words verbatim) 2. Clarity & suitability of aim for meta-ethnography (describe in words) 3. Focus of review   a. Disease or clinical condition/health issue Y/N  b. Health services or technologies e.g. health promotion, intervention Y/N  c. Other health and/or social topics Y/N  **Phase 2**   1. Sampling of papers: did they use exhaustive searches, purposive sampling etc. (describe in words)? 2. Comprehensive for/no limits on date range of searches? Y/N 3. Did they give a clear description of: 4. Databases used? Y/N 5. Supplementary search strategies e.g. hand searching of journals, contacting experts, reference list searches? Y/N   c. Key words/ search terms? Y/N   1. Number of papers/studies included in review 2. a. Was a method of critical appraisal of papers used? Y/N   b. Method of critical appraisal  **Phases 3-6**  14 Which key meta-ethnography methodological papers do they cite?  Noblit & hare 1988 Y/N  Campbell et al 2003 Y/N  Campbell et al 2011 Y/N  Britten et al 2002 Y/N  Atkins et al 2008 Y/N  Malpass et al 2009 Y/N  15 Conflict of interest Y/N/Not stated  16 Funders – specify or say not stated   1. Quality of analysis and synthesis reporting   17a. Do they describe clearly N&H's three analytical phases 4-6 (with more than the brief label for each phase)? Y/N  17b. How many researchers were involved in analysis? Y/N  **Phase 3**  17c. Do they state the order in which papers were read and synthesised? Y/N  17d. Do they describe clearly how they identified concepts/themes/ metaphors from papers? Y/N  **Phase 4**  17e. Did they state that they carried out phase 4- Determining how the studies are related? Y/N  17f. Did they clearly describe how they did phase 4- Determining how the studies are related? Y/N  17g. Do they report the total no. of 2nd order constructs/ themes/ concepts/ metaphors found in included papers? Y/N  **Phases 5 & 6**  17h. Do they describe clearly how they did the reciprocal or refutational translation process? Y/N  17i. Do they describe clearly how they synthesised concepts ('translations')? Y/N  17j. Do they describe clearly which papers contributed to each final (third order) construct? Y/N  17k. Do they present a new interpretation (model, lines-of-argument synthesis or third order construct)? Y/N  17l. Do they claim to present third order constructs (even if labelled differently)?  17m. Do they claim to present a line-of-argument synthesis?  17n. Do they claim to present a new model or theory?  **Phase 7**  17o. How did they express the synthesis (phase 7) - text only (can include tables), text + pictorial/visual model?  17p. Did they present 1st order constructs, i.e. participant quotes, in the findings section?  17q. Is what they did recognisable as a meta-ethnography? Y/N  18. Comments & details (free text):  19. Reviewer initials: |
| --- |

**Key: Y=yes, N=no**
