# Supplementary material for: A methodological systematic review of what’s wrong with meta-ethnography reporting
Source: BMC Med Res Methodol. 2014 Nov 19;14:119. doi: 10.1186/1471-2288-14-119 (PMC4277825; doi:10.1186/1471-2288-14-119)
Supplement: Supplementary file 4 — Additional file 4: Table S2: Summary of characteristics of papers included in the review. (DOCX 24 KB) [file 12874_2014_1138_MOESM4_ESM.docx]

Table S2. Summary of characteristics of papers included in the review

| **Characteristic/issue** |  | **N (%) of papers** |
| --- | --- | --- |
| **Methodological terms used in paper title:*** | Includes term ‘meta-ethnography’ | **10** (31%) |
|  | Includes term ‘meta-ethnographic’ | **3** (9%) |
|  | Includes term ‘meta-synthesis’ | **9** (28%) |
|  | Includes term ‘systematic review’ | **10** (31%) |
|  | Uses other terms (qualitative synthesis, synthesis) | **2** (6%) |
|  | None of above terms | **3** (9%) |
| **Methodological terms used in paper abstract:*** | ‘Meta-ethnography’ | **18** (56%) |
|  | ‘Meta-ethnographic’ | **9** (28%) |
|  | ‘Meta-synthesis’ | **5** (16%) |
|  | ‘systematic review’ | **3** (9%) |
|  | None of above terms | **2** (6%) |
| **Focus of article:** | Disease or clinical condition or health issue | **12** (38%) |
|  | Health services or technologies including interventions | **10** (31%) |
|  | Other health or social topic | **10** (31%) |
| **Journals published in:** | Midwifery/pregnancy | **7** (22%) |
|  | Nursing | **6** (19%) |
|  | Psychiatry/psychotherapy/psychology | **4** (%) |
|  | Public health | **2** (6%) |
|  | Other | **12** (%) |
| **Phase 2. ‘Describing what is relevant to initial interest’** | | |
| **Search methods:** | Exhaustive sampling | **26** (81%) |
|  | No literature search conducted | **1** (3%) |
|  | Insufficient information to determine sampling methods | **4** (13%) |
| **Search methods:** | Comprehensive date range | **8** (25%) |
|  | Date range not stated | **4** (13%) |
|  | Gave clear description of databases used | **24** (78%) |
|  | Used supplementary search strategies | **21** (66%) |
|  | Gave clear description of key words/search terms | **21** (66%) |
|  |  |  |
|  | Number of included studies | Range 3-60, mean=20, median=18 |
|  | Number of included papers | Range 3-77, mean=21, median=18 |
| **Critical appraisal:** | Used a method of critical appraisal | **26** (81%) |
|  | CASP method | **12** (38%)* |
|  | COREQ method | **2** (6%)* |
|  | JBI QARI method | **2** (6%)* |
|  | Other method of critical appraisal | **12** (38%)* |
| **Quality of analysis and reporting:** | Clearly described Noblit and Hare’s analytic phases of meta-ethnography (with more than just the labels) | **11** (34%) |
|  |  |  |
|  | Clearly recognisable as meta-ethnography | **11** (34%) |
|  | Not recognisable as meta-ethnography | **14** (44%) |
|  | Unclear from information provided whether synthesis was a meta-ethnography | **7** (22%) |
| **Phase 3. ‘Reading the studies’** | | |
|  | Stated order in which papers were read/synthesised | **5** (16%) |
|  | Clearly described how concepts/metaphors were identified | **23** (72%) |
|  | Reported total number of second order constructs | **9** (28%) |
| **Phase 4. ‘Determining how studies are related’** | | |
|  | Stated that they carried out phase 4- Determining how the studies are related | **26** (81%) |
|  | clearly described how they did determined how the studies are related | **21** (66%) |
| **Phase 5. ‘Translating the studies into one another’** | | |
|  | Clearly described reciprocal/refutational translation process | **10** (31%) |
| **Phase 6. ‘Synthesizing translations’** | | |
|  | Clearly described how concepts/translations were synthesised | **1** (3%) |
|  | Clearly described which papers contributed to new interpretations | **14** (44%) |
|  | Claimed to present third order constructs (even if labelled differently) | **15** (47%) |
|  | Claimed to present a line-of-argument synthesis | **17** (53%) |
|  | Claimed to present a new conceptual model or theory | **10** (31%) |
|  | Presented a new interpretation | **13** (41%) |
|  | Did not present new interpretation | **7** (22%) |
|  | Unclear from information given if presented new interpretation | **12** (38%) |
| **Number of researchers carrying out analysis** | Not stated / not clear | **9** (28%) |
|  | 1 | **5** (16%) |
|  | 2 | **6** (19%) |
|  | 3+ | **12** (38%) |
| **Authors’ conflict of interest/competing interests** | States no conflict of interest | **21** (66%) |
|  | States conflict of interest | **1** (3%) |
|  | Not stated | **11** (34%) |
| **Phase 7 – ‘Expressing the synthesis’** | | |
|  | Written format of findings only | **16** (50%) |
|  | Written and visual representations of findings | **16** (50%) |
|  | Present participant quotes from primary studies in findings | **26** (81%) |
|  | Present quotes from the authors of primary studies in findings | **1** (3%) |
| **Key methodology texts cited:*** | Noblit, G. W., & Hare, R. D. (1988). Meta–Ethnography: Synthesizing Qualitative Studies (1988). *Stage, Newbury Park, CA*. | **32** (100%) |
|  | Britten, N., Campbell, R., Pope, C., Donovan, J., Morgan, M., & Pill, R. (2002). Using meta ethnography to synthesise qualitative research: a worked example. *Journal of Health Services Research & Policy*, *7*(4), 209-215. | **10** (31%) |
|  | Campbell, R., Pound, P., Pope, C., Britten, N., Pill, R., Morgan, M., & Donovan, J. (2003). Evaluating meta-ethnography: a synthesis of qualitative research on lay experiences of diabetes and diabetes care. *Social science & medicine*, *56*(4), 671-684. | **10** (31%) |
|  | Campbell, R., Pound, P., Morgan, M., Daker-White, G., Britten, N., Pill, R.,Yardley, L., Pope, C. & Donovan, J. (2011). Evaluating meta ethnography: systematic analysis and synthesis of qualitative research. *Health Technology Assessment*, *15*(43). | **7** (22%) |
|  | Atkins, S., Lewin, S., Smith, H., Engel, M., Fretheim, A., & Volmink, J. (2008). Conducting a meta-ethnography of qualitative literature: lessons learnt. *BMC medical research methodology*, *8*(1), 21. | **8** (25%) |
|  | Malpass et al 2009 Malpass, A., Shaw, A., Sharp, D., Walter, F., Feder, G., Ridd, M., & Kessler, D. (2009). “Medication career” or “Moral career”? The two sides of managing antidepressants: A meta-ethnography of patients' experience of antidepressants. *Social science & medicine*, *68*(1), 154-168. | **7** (22%) |

*Some papers are counted in more than one category.
