# Supplementary material for: A methodological systematic review of what’s wrong with meta-ethnography reporting
Source: BMC Med Res Methodol. 2014 Nov 19;14:119. doi: 10.1186/1471-2288-14-119 (PMC4277825; doi:10.1186/1471-2288-14-119)
Supplement: Supplementary file 5 — Additional file 5: Table S3: Characteristics of individual included papers and reporting of meta-ethnography Phases 1 and 2. (DOCX 38 KB) [file 12874_2014_1138_MOESM5_ESM.docx]

Table S3. Characteristics of individual included papers and reporting of meta-ethnography Phases 1 and 2

|  |  |  | |  | | **Main focus of review** | | | | | | **Sampling of papers** | | | | **Search strategy/method** | | | | | |  | | **Critical appraisal** | | |
| --- | --- | --- | --- | --- | --- | --- | --- | --- | --- | --- | --- | --- | --- | --- | --- | --- | --- | --- | --- | --- | --- | --- | --- | --- | --- | --- |
| **Paper** | **Aim** | **COI** | | **Funders** | | **Disease or clinical condi-tion/**  **health issue** | | **Health services/ technologies e.g. health promotion, intervention** | | **Other health and/or social topics** | | **Used exhaustive searches** | | **Compreh-ensive date range for searches? Y/N** | | **Clear description of databases used?** | | **Used supple-mentary search strategies** | | **Clear description of key words/ search terms?** | | **No. of papers/**  **studies included in review** | | **Used method of critical appraisal** | | **Method of critical appraisal (CASP, COREQ, JBI QARI etc.)** |
| S1 | ‘This meta-ethnography aims to give the deeper insight needed into nurse-patient relationships by synthesizing research that explores the experiences of nurses in these relationships. The objectives were: To understand how nurses characterize their relationships with adult patients in acute inpatient hospital settings. To understand the strategies that nurses use to build and sustain relationships with patients. To understand the impact on nurses for being in the nurse-patient relationship. To identify the factors that inﬂuence the relationships between nurses and patients. The focus on adult patients reﬂected a wish to better understand the factors associated with reported care failures in adult settings’ P. 761 | - | | NHS trusts: Barnet and Chase Farm Hospitals NHS Trust and Royal Free Hampstead NHS Trust, UK | | - | |  | | - | |  | | - | |  | |  | |  | | 18 | |  | | CASP |
| S2 | ‘This study aims to determine the following: 1. whether people with personal experience of a mental health problem believe that disclosing this will lead to unfavourable treatment in employment’ P.P. 2 | - | | National Institute for Health Research (NIHR) Programme Grants for Applied Research scheme & the Trustees of South London and Maudsley NHS Foundation Trust, UK. | |  | | - | | - | |  | | - | |  | |  | |  | | 17 studies | |  | | other |
| S3 | ‘This meta-synthesis aims to gain a deeper understanding of how the features of Mindfulness-based Cognitive Therapy contribute to positive therapeutic change.’ P.1  ‘the focus of this study was upon the impact of the therapy upon participants’ parenting skills rather than the experiences of the programme itself’ P. 2 | NS | | NS | | - | |  | | - | |  | |  | |  | |  | |  | | 7 | |  | | CASP |
| S4 | ‘the aim of this review is to identify key factors that act as barriers and facilitators to the effective implementation of evidence-based best practice in relation to the prevention of falls among community-dwelling older people.’ P. 2 | - | | NIHR Collaboration for Leadership in Applied Health Research and Care (CLAHRC) for the South West of England. Part funded by European Regional Development Fund and the European Social Fund Convergence Programme for Cornwall and the Isles of Scilly, UK | | - | |  | | - | |  | | - | |  | | - | | - | | 19 | |  | | other |
| S5 | ‘Here we report a meta-ethnography on the perspectives of paid dementia care providers on the jobs they do, present the overarching themes uncovered, and discuss the implications of these findings for dementia care.’ P. 127 | - | | Canadian Institutes for Health Research | | - | | - | |  | |  | | - | |  | | - | |  | | 34 | |  | | other |
| S6 | ‘We ﬁnd the differences between labouring women’s perceived needs and health-care providers’ ability to care for lesbian women disturbing. We therefore set up a study to explore and synthesize qualitative research knowledge about lesbian women’s experiences with health-care providers in the birthing context.’ P. 675 | - | | The Norwegian Women’s Public  Health Association | | - | |  | | - | |  | |  | |  | |  | |  | | 13 | |  | | other |
| S7 | ‘Purpose: To synthesize published qualitative studies concerning the lived experience of rheumatoid arthritis (RA). To compare the conceptual features of qualitative studies covering two different time periods.’ P. 1 | - | | NIHR Health Technology Assessment Programme, UK (first synthesis only, second was unfunded) | |  | | - | | - | |  | | - | |  | |  | | - | | 1) 21 studies in 24 papers; 2) 28 papers | |  | | CASP |
| S8 | ‘the aim of this systematic review is to pro- vide in-depth consideration for the subjective view of COPD [chronic obstructive pulmonary disease] patients on the impact of PR [pulmonary rehabilitation] on their lives, exploring the lived experiences and identifying the benefits, negative and positive aspects perceived during and/or after participation in PR programmes.’ P. 142 | - | | Ministerio de Asuntos Exteriores (MAE), Spain | | - | |  | | - | |  | | - | |  | | NS | |  | | 8 | |  | | other |
| S9 | ‘ Our aim was to synthesise qualitative literature about the perceived impact and experience of participating in peer support interventions for individuals with chronic disease.’ P. 3  ‘This paper synthesizes qualitative research about the experiences and perceived impacts of peer support interventions across multiple chronic diseases, and in so doing, works towards a conceptual model. It also aims to identify both positive and negative aspects of peer support, and examine which experiences and perceived impacts have relevance for mentors and mentees.’ P. 4 | NS | | Canadian Institutes for Health Research &  Ontario Rehabilitation Research Advisory Network, Canada & partially supported by NIHR CLAHRC for the South West Peninsula, UK. | | - | |  | | - | |  | |  | |  | |  | | - | | 25 | |  | | other |
| S10 | ‘The goal of the project was to describe the concepts, expectations and perceptions of individualised medicine inherent in patients’ reasons for using CAM [complementary and alternative medicine], as documented in qualitative studies.’ P. 2 | - | | NS | | - | | - | |  | |  | | - | | - | | - | |  | | 30 | |  | | NOT STATED |
| S11 | ‘With the aim to identify how women's experiences of health services following severe maternal morbidity could be improved, we explored women's perceptions and experiences of severe maternal morbidity (deﬁned as major obstetric haemorrhage, severe preeclampsia, eclampsia, HELLP syndrome, critical care unit admission) by synthesising evidence from qualitative studies.’ P. 1 | NS | | No funding | |  | | - | | - | |  | | - | |  | |  | |  | | 12 | |  | | CASP |
| S12 | ‘The aim of this study was to report the results of a metasynthesis of qualitative studies on nursing leadership development and to enhance an understanding of both those conditions nurses have reported to be effective and supportive, as well as those that have hindered their development as leaders in nursing.’ P. 333 | - | | NS | | - | | - | |  | | DK | | - | | - | |  | | - | | 21 | | NS | | NS |
| S13 | ‘This study had two aims: (i) to utilize the ﬁndings of published qualitative research, speciﬁcally related to neurological conditions, in order to develop a deeper understanding of the impact of cognitive impairment on the carer and to ﬁnd areas of concordance across four of the most common neurological diseases; and (ii) to provide a worked example of a meta-ethnography’ P. 114 | NS | | NS | | Y | | - | | - | |  | | - | |  | |  | |  | | 31 | |  | | Dixon-Woods et al., 2001 CRITERIA |
| S14 | ‘The aim of this study was to synthesise qualitative literature to describe how immigrant women experience maternity services in Canada.’ P. 2 | NS | | Faculty of Medicine and Dentistry, University of Alberta, the Women and Children's Health Research Institute &  Alberta Health Services, Edmonton, Canada & a Canada Research Chair in Ethnicity and Health | | - | |  | | - | |  | | - | |  | |  | | - | | 22 | |  | | JBI QARI |
| S15 | ‘Objective: To synthesize evidence of family members recognizing that their relative is likely to die within the year, and identifying the need for palliative care. ‘ P.108  ‘Relatives’ views on prognosticating may be one method of augmenting the clinical judgements of experienced physicians and nurses and thereby improving prognostication. However, there are no published systematic reviews that have reported on the existing evidence in this important area and there appear to be few studies that have addressed it.  The aim of the review was therefore to improve understanding of experiences of family members by systematically searching for papers that report experiences of family members of people with PD [Parkinson’s disease], MND [motor neurone disease] and MS [multiple sclerosis] recognizing that their relative was ‘likely to die within the year’.’ P. 109 | NS | | NHS Lanarkshire Health Board, Scotland | |  | | - | | - | |  | | - | |  | | NA | |  | | 9 | | - | | NA |
| S16 | ‘This review reports the first meta-ethnography to address the question: through what processes does the school environment (social and physical) influence student health outcomes?’ P. 2 | - | | NIHR Public Health Research Programme, UK | | - | | - | |  | |  | | DK | |  | | NS | | - | | 19 | |  | | criteria from EPPI-Centre |
| S17 | ‘The aim of this meta-synthesis is to examine what is known about patients' experiences of HNC [head and neck cancer] by drawing together the ﬁndings of existing qualitative studies to provide a context for future research.’ P. 2649 | NS | | Chief Scientist Office (Scottish Government) & Macmillan Cancer Support charity, UK | |  | | - | | - | |  | |  | |  | | - | | - | | 29 | |  | | CASP but adapted |
| S18 | ‘There is a need to integrate qualitative findings of women’s experiences of VBAC [vaginal birth after caesarean] to influence evidence-based practice but also to generate new research questions. The objective of this metasynthesis is therefore to inte- grate the findings and deepen the understanding of women’s experiences of VBAC.’ P. 2 | - | | NS | |  | | - | | - | |  | | DK (say no studies found before 2002) | |  | |  | |  | | 8 | |  | | COREQ plus other items |
| S19 | ‘The aim of the present study was to analyse, through an interpretative systematic review of qualitative studies, the meaning and motivation of the WTHD [wish to hasten death] in patients with chronic illness or advanced disease.’ P. 2 | - | | Collegi Oficial d’Infermeria de Barcelona and a National Grant from Instituto de Salud Carlos III, Spain | | - | | - | | Y | |  | |  | |  | |  | |  | | 7 | |  | | CASP |
| S20 | ‘While qualitative studies have been undertaken to investigate factors influencing uptake of HIV testing, systematic reviews to provide a more comprehensive understanding are lacking.’ P. 1  ‘Qualitative studies have also been conducted in SSA that additionally highlighted social dynamics influencing uptake of HIV testing. Despite the volume of this evidence and the contribution it can make towards a better understanding of factors influencing uptake of HIV testing in SSA, systematic reviews are lacking.’ P. 2 | - | | Swiss National Science Foundation | |  | | - | | - | |  | | DK | |  | |  | |  | | 42 | | - | | NA |
| S21 | ‘The purpose of this metasynthesis was to synthesize the ﬁndings of qualitative research studies related to infant feeding decision making and contribute to an increased understanding of this phenomenon.’ P.247  ‘The purpose of the current metasynthesis was to add to these previous efforts by focusing more narrowly on understanding maternal infant feeding decision making through synthesizing ﬁndings from a group of 14 qualitative studies on this topic.’ P. 248 | - | | NS | |  | | - | | - | | DK | | - | |  | |  | |  | | 14 | | NS | | NS |
| S22 | ‘the aim of this meta-ethnographic synthesis was to describe the physiological and psychological experiences of women who have experienced severe perineal trauma so as to inform the practice of health professionals specializing in the care of childbearing women, particularly during the postnatal period.’ P. 749 | - | | No funding | |  | | - | | - | |  | | - | |  | |  | |  | | 4 | |  | | CASP |
| S23 | ‘Aim To explore and interpret how nursing students develop their understanding of the patient as a human being.’ P. 771 | NS | | University West | | - | | - | |  | |  | | - | |  | | NS | |  | | 17 | |  | | CASP |
| S24 | ‘The aim of this meta-ethnographic study was to explore the diversity of migrant and refugee women’s experiences and practices related to breastfeeding in a new country with a view to informing health care policy and practice and identifying ways in which migrant and refugee women can be better supported to achieve their breastfeeding goals.’ P. 2 | - | | NS | | - | | - | |  | |  | | - | |  | |  | | - | | 11 | |  | | CASP |
| S25 | ‘Objective: To synthesise the existing published literature on the perceptions of general practitioners (GPs) or their equivalent on the clinical management of multimorbidity and determine targets for future research that aims to improve clinical care in multimorbidity.’ P. 1 | √ | Funding Health Research Board, Ireland | | - | | - | |  | |  | |  | |  | |  | |  | | 11 | |  | | CASP | |
| S26 | ‘This study speciﬁcally aimed to assess the literature to determine: what are the experiences and attitudes of people towards the conservative management of osteoarthritis?’ P. 2 | - | | NS | | Y | | - | | - | |  | |  | |  | |  | |  | | 33 | |  | | CASP & Gough weight of evidence appraisal tools |
| S27 | ‘The UK National Childbirth Trust (NCT) has recommended that research is needed to assess how health professionals engage with expectant and new fathers, and to examine the views and experiences of men in this context. As a ﬁrst step in the process, this paper presents the ﬁndings of a metasynthesis of good quality qualitative research undertaken in this area, with the aim of exploring the views and experiences of fathers who have encountered maternity care in high resource contexts over the last 10 years.’ P. 423 | NS | | Central Manchester University Hospitals  NHS Foundation Trust, UK | | - | | - | |  | | NS | | - | |  | |  | |  | | 23 studies | |  | | other |
| S28 | ‘The aim of this review was to synthesise existing qualitative research to improve understanding and thus best practice for people with chronic non-malignant MSK pain.’ P. e829 | - | | NIHR Health Services and Delivery Research Programme, UK | |  | | - | | - | |  | |  | |  | |  | |  | | 77 papers from 60 studies | |  | | 3 method: CASP JBI QARI; & papers assessed for concept-ual richness |
| S29 | ‘The aim of this study was to develop a theory model concerning crucial aspects inherent in dignity-preserving dementia care as perceived and practiced among nurses and allied HCP documented in previous empirical qualitative studies. Its purpose was the development of a unique understanding concerning dignity- preserving dementia care, making such knowledge available to practitioners, scholars, and politicians responsible for quality dementia care promotion.’ P. 2 | - | | Oslo and Akershus University College (doctoral funding), Norway | | - | |  | | - | |  | | - | |  | |  | | - | | 10 | |  | | COREQ |
| S30 | ‘we know that EBF [exclusive breast feeding] is vital for the health of HIV-exposed infants, and that rates of EBF are low in SSA [sub-Saharan Africa], but our understanding of why is limited. Therefore, we conducted a metasynthesis to enhance our understanding of the collective experiences of infant feeding with HIV' mothers in SSA.’ P. 215 | NS | | National Institute of Mental Health, Unites States of America | | - | | - | |  | | DK | | - | |  | | NS | | - | | 16 | | - | | NA |
| S31 | ‘This article responds to the need to document interventions to integrate HIV care into primary care services, to record outcomes of these interventions and to provide analytical insights to inform both programme management and further theoretical development of the area. We report on factors perceived to influence the integration of HIV care into primary care services at the level of service delivery during a randomised controlled trial of strategies to improve access to ART in South Africa: the Streamlining Tasks and Roles to Expand Treatment and Care for HIV (STRETCH) trial. Lessons learnt in the area of integration of HIV care may be useful in informing broader questions regarding the integration of programmes into service delivery in primary care.’ P. 2 | - | | International Development Research Centre (IDRC), Canada, British Medical Research Council & Irish AID & National Research Foundation, South Africa | | - | |  | | - | | - | | - | | - | | - | | NA | | 3 | | - | | NA |
| S32 | ‘reviews of return to work in cancer have, so far, only focussed on breast cancer. We therefore conducted a meta-synthesis of qualitative studies to elucidate the complex issues surrounding returning to work after cancer and to develop a robust theoretical and empirical basis for the development of future interventions. The following questions guided the review:  (1) What are cancer survivors’ (a) attitudes to work during and after cancer treatment? (b) experiences (both positive and negative) of gaining employment, working through treatment or returning to work? (c) strategies to overcome any challenges experienced?  (2) What are the roles, attitudes and experiences of family/ carers’ and/or employers’ in relation to facilitating or obstructing cancer survivors’ work experiences?’ P. 1209 | NS | | not stated | | - | |  | | - | |  | | NS | |  | |  | |  | | 25 | | Y | | CASP & COREQ |

Key: ‘-’ = no, √ = yes, NS = not stated, NA = not applicable, DK = don’t know (not enough detail given), COI= conflict of interest
