# Supplementary material for: A methodological systematic review of what’s wrong with meta-ethnography reporting
Source: BMC Med Res Methodol. 2014 Nov 19;14:119. doi: 10.1186/1471-2288-14-119 (PMC4277825; doi:10.1186/1471-2288-14-119)
Supplement: Supplementary file 6 — Additional file 6: Table S4: Reporting of meta-ethnography analytic phases 3 to 7 in included papers. (DOCX 29 KB) [file 12874_2014_1138_MOESM6_ESM.docx]

Table S4. Reporting of meta-ethnography analytic phases 3 to 7 in included papers

| **Paper** | **Describe fully Noblit & Hare's 7 phases** | **Phase 3. State order of reading & synthesising papers** | **Phase 3. State how they identified concepts/**  **themes from primary studies** | **Phase 4. State they deter-mined how the studies (papers) are related?** | **Phase 4. Clearly describe how they deter-mined how the studies (papers) are related?** | **Phase 5. Clearly describe translation process** | **Phases 5&6. Number researchers involved in analysis** | **Phase 6. Clearly describe how synthesised translations** | **Phase 6. State which papers contributed to each new concept /inter-pretation** | **Phase 6. Present new inter-pretation of concepts** | **Phase 6. (Claim to) present 3rd order construct** | **Phase 6. (Claim to) present line of argument synthesis** | **Phase 6. (Claim to) present new model or theory?** | **Phase 7. How did they express synthesis?** | **Phase 7. Gave part-icipant quotes in findings** | **Phase 7. Recognisable as a meta-ethnography?** |
| --- | --- | --- | --- | --- | --- | --- | --- | --- | --- | --- | --- | --- | --- | --- | --- | --- |
| S1 |  | - |  |  | - |  | 1 | - |  |  |  |  | - | text only |  |  |
| S2 | - | - | - | - | - | - | 1 | - |  | - | - | - | - | text only |  | - |
| S3 | - | - |  |  |  | - | 1 | - | - | DK |  | - | - | both text & visual |  | DK |
| S4 | - | - | - | - | - | - | 2 | - |  | DK | - |  | - | text only |  | DK |
| S5 |  | - |  |  |  | - | 1 | - | - | DK |  |  | - | both text & visual | - | DK |
| S6 |  | - |  |  | - | - | 4 | - | - |  |  | - | - | text only |  |  |
| S7 | - |  |  |  |  | - | 5 in study one, 1 in study two | - |  | - | - | - | - | both text & visual |  | - |
| S8 | - | - |  |  | - | - | 3 | - | - | DK |  | - | - | text only |  |  |
| S9 | - | - |  | - | - | - | 3 to 4 | - |  |  |  |  |  | both text & visual | DK |  |
| S10 |  |  |  |  |  |  | 2 |  | - |  | - |  | - | both text & visual |  |  |
| S11 |  | - |  |  |  | - | NS | - | - | DK | - | - | - | both text & visual |  | DK |
| S12 |  | - |  |  |  |  | NS (only 1 author) | - | - | DK |  | - | - | text only |  | - |
| S13 |  | - |  |  |  | - | 5 | - | - | DK | - |  |  | both text & visual |  | - |
| S14 | - | - |  |  |  | - | 1 main | - |  |  |  |  | - | both text & visual | - |  |
| S15 |  | - |  |  | - | - | 2 | - |  | - | - | - | - | text only |  | - |
| S16 |  | - |  |  |  |  | 2 | - | - |  |  |  |  | both text & visual |  | - |
| S17 | - | - |  |  |  |  | 4 to 5 | - | - |  | - | - |  | both text & visual |  |  |
| S18 | - | - |  |  |  | - | NS | - | - | DK | - | - | - | text only |  | DK |
| S19 | - |  |  |  |  |  | 1 main with 4 others involved | - |  |  | - |  |  | both text & visual |  |  |
| S20 | - | - | - |  |  |  | NS | - |  |  |  |  |  | both text & visual |  | - |
| S21 | - | - | - |  | - | - | NS (only 1 author) | - | - | DK | - | - | - | text only |  | - |
| S22 | - | - |  |  |  |  | 1 main with 2 others involved | - | - |  |  |  | - | text only |  | DK |
| S23 | - | - | - |  |  | - | 2 | - | - | - | - |  | - | text only | - | - |
| S24 | - | - | - |  |  | - | 2 | - | - | - | **-** | - | - | text only |  | - |
| S25 |  |  |  |  |  |  | 3 | - |  |  |  |  | - | text only |  |  |
| S26 | - | - | - | - | - | - | 3 | - | - | DK | - |  |  | both text & visual | - | - |
| S27 | - | - | - | - | - | - | DK | - | - | - | - | - | - | text only |  | - |
| S28 | - | - |  | - | - | - | 3 | - |  | DK | - |  |  | both text & visual |  | - |
| S29 |  | - |  |  |  | - | 3 | - |  |  |  |  |  | both text & visual |  | - |
| S30 | - | - | - |  |  | - | NS | - | - | - | - | - | - | text only |  | - |
| S31 | - |  |  |  |  | - | 3 | - |  | DK | - | - | - | text only |  | - |
| S32 | - | - |  |  |  |  | 7 | - |  |  |  |  |  | both text & visual | - |  |

Key: ‘-‘ = no, √ = yes, NS = not stated, DK = don’t know (not enough detail given)
