# Supplementary material for: A methodological systematic review of what’s wrong with meta-ethnography reporting
Source: BMC Med Res Methodol. 2014 Nov 19;14:119. doi: 10.1186/1471-2288-14-119 (PMC4277825; doi:10.1186/1471-2288-14-119)
Supplement: Supplementary file 7 — Additional file 7: Table S5: Illustrative examples of analysis and synthesis process reporting in papers included in review. (DOCX 30 KB) [file 12874_2014_1138_MOESM7_ESM.docx]

Table S5. Illustrative examples of analysis and synthesis process reporting in papers included in review

| **Topic/issue** | **Examples of reporting lacking transparency** | **Examples of transparent reporting** |
| --- | --- | --- |
| **Reporting of phase 5 - the reciprocal/ refutational translation process**  **Reporting of phase 5 - the reciprocal/ refutational translation process**  **Reporting of phase 5 - the reciprocal/ refutational translation process**  **Reporting of phase 5 - the reciprocal/ refutational translation process**  **Reporting of phase 5 - the reciprocal/ refutational translation process**  **Reporting of phase 5 - the reciprocal/ refutational translation process** | **Paper S2, p.3.** ‘Data were managed using NVivo version 8 [27]. The results and discussion sections of each study were entered into NVivo, and verbatim examples from each study were coded using the original codes suggested by the authors. A master list of all codes used was produced and examined for connections. The examples from each study were then coded again using a reduced list of codes. After all transcripts had been coded in this way the master list was reviewed again and structuring of super-ordinate themes and sub-themes were considered. All studies were then coded using the revised list of super-ordinate themes and sub-themes. Each sub-theme was present in at least two studies, while a minimum of one of the sub-themes of each super-ordinate theme was endorsed by 50% or more of studies (8/17 studies). In this way, the super-ordinate themes and sub-themes were reflective of the included studies.’  **Paper S4, p. 2.**  ‘While reading and rereading the papers, we used thematic analysis to identify recurrent issues arising in the studies. We extracted quotes relating to barriers and facilitators to fall-prevention interventions, which were later tabulated in order to develop first- and second- order concepts from each paper.’  **Paper S6, p.675.** ‘The interpretive metaphors as expressed by the primary authors (content issues, themes, concepts, phrases) were noted and processed into a grid listing studies horizontally and interpretive metaphors vertically […] The grid enabled us to compare related metaphors or content issues from the studies (step 5), e.g. ‘fear of being looked upon as ‘bad mothers’, ‘fear of homophobia’, ‘fear for refusal of rights’ and ‘coming out carrying enormous personal risk’. Further, we synthesized issues from the same row into a common concept by reciprocal translation (step 6).’  **Paper S11, p. 4.** ‘Translating one study's ﬁndings into another. This process involved further comparison of the second order constructs across studies, examining similarities and interactions between them (Noblit and Hare, 1988). The process of translation was aided by listing the key themes and concepts in a table with the authors' own words or participants' own words when necessary.’  **Paper S12, p. 335**  ‘Translating is an interpretive process that preserves the integrity of the initial account of the phenomenon and the metaphors used to describe it but translates those metaphors into those identiﬁed in the other studies. Processes in phases 4 and 5 yielded 3 overarching themes or metaphors.’  **Paper S23, p. 773**  ‘As Noblit and Hare (1988) explained: ‘Translations are especially unique syntheses, because they protect the particular, respect holism and enable comparison’ (p. 28). This phase involved combining the studies, i.e. translating them into one another by comparison. The translation must protect the speciﬁc and maintain the wholeness (Noblit & Hare 1988).’  **Paper S29, pp. 4 & 8**  ‘Translating the studies into one another: Translating second-order constructs, developing third-order constructs (first-level synthesis).’  ‘In order to preserve wholeness, we found reasons to bring the synthesis process forward, utilizing reciprocal translation of the studies into one another in Groups A and B separately, before drawing them together in a final lines-of-argument synthesis.’  **Paper S30, p. 215.**  ‘Nobit [*sic*] and Hare (1988) identify three assumptions upon which studies may be related, i.e., reciprocally, through illustration of a line of argument or refutational/opposition. We use their first assumption, ‘‘that the accounts are directly comparable as ‘reciprocal’ translations’’’. We then translated the key metaphors of each study into more general terms that fit across all studies.’  **Paper S31, p. 4**. ‘the primary (participant understanding) and secondary (researcher interpretation) themes from the individual studies were presented, and common categories across these themes were identified. These were iteratively refined through further discussion into comprehensive categories and sub-categories. Translating the studies into each other involved developing these categories, with detailed data from each study added and used to elucidate and elaborate these common categories. Table 3 gives an illustrative view of this process’ | **Paper S1, pp. 762-763**  ‘Synthesis began with repeated readings of the studies to identify key categories and to determine relationships between individual studies. A list of key categories was thus generated and used as the basis for comparing and sorting interpretations, examining similarities and differences, and then integrating these in a new (‘third order’) interpretation that applies across the studies, referred to as a ‘line-of-argument’ (Noblit & Hare 1988, Britten et al. 2002, Pope et al. 2007). […]  For this synthesis, second-order interpretations were extracted against the list of key categories identiﬁed and these were used as a foundation for exploring translations between the studies. Much of the detail of the second-order interpretation was retained at this stage, to help preserve context and meaning. Comparisons were then made across the studies to determine the extent to which concepts proposed in one study related to those expressed in another study, a process known as reciprocal translation (Noblit & Hare 1988). Differences were pursued as rigorously as similarities and comparisons across concepts and contexts were continuously made by, for instance, exploring the extent to which an emerging interpretation was relevant across clinical settings. The translated concepts were then used to identify third-order interpretations that transcended the individual accounts (Pope et al. 2007). As third-order interpretations emerged, they were systematically tested by looking across all the studies and the second-order interpretations; these third-order interpretations were discarded or developed further as required. Reciprocal translation continued until no further third-order interpretations emerged.’  **Paper S10, p.4**  ‘Translation in a meta-ethnography such as ours means comparing the metaphors and concepts in one article with the metaphors in others. We first arranged all papers chronologically and according to main indications. Thereafter, we compared the key themes from paper one with paper two, and the syntheses of these two papers with paper three, and so on. The translation respected the individual meaning and maintained the central metaphors in relation to the studies’ other key metaphors. We translated our key themes across all articles in order to determine secondary key themes. All secondary key themes contributed reasoning behind why patients turn toward CAM [complementary and alternative medicine]. To perform the translation, the research team members worked with grids or hand cards. The relationship between the studies was indicated by drawing arrows, lines and bubbles or by clustering the hand cards. The emerging secondary key themes were transferred into the head line of a spreadsheet named “secondary key themes,” and the applicable explanations were entered in the rows below, the themes were juxtaposed with the authors’ main secondary interpretations from the discussion section of each article. We made analytical and reflexive notes during the translation to be prepared for the research group discussions.’  **Paper S19, p. 4**  ‘Having identified the main concepts that emerged from each paper, a search was then undertaken for the presence or absence of these concepts in the seven papers. During this process the authors made sure that each key concept took on similar meanings in all the papers, although they also identified those which were unique or specific to one or more of the studies. […] The comparison process began with the themes identified in the first study, to which others were added as they emerged. At the same time, these themes were newly translated to the whole sample and to each individual study. The reciprocal translations enabled us to develop a table that shows each theme with its categories, as well as quotations from participants to explain each theme (see Table 3).’  **Paper S20, p.3.** ‘To establish how the concepts from different papers were related to one another, we created a grid and entered the concepts from each paper […].We used Malpass’s notion of first-, second-and third-order constructs to generate the concepts [26]. First-order constructs represent the views of research participants while second order-constructs are authors’ interpretation of research participants’ views [14,23]. Using the process of translation – transfer of ideas, concepts and metaphors across different studies [14], we compared the concepts of the papers i.e. paper 1 with paper 2 and the synthesised concepts of the two papers with paper 3 and so on, until all studies had been translated into each other [16,23].’  **Paper S22 pp. 751-752**  ‘Data abstraction and synthesis.  Using the methods of reciprocal translation and refutational investigation, ﬁve initial themes were identiﬁed during the initial synthesis undertaken by the ﬁrst author. This initial process involved the design of a table which listed each major thematic ﬁnding of the four included papers. The ﬁrst author then worked through these themes to identify any similar (reciprocal translation) or contrasting (refutational investigation) themes or metaphors across the articles (Noblit & Hare 1988). No refutational ﬁndings were identiﬁed between the four studies. From this initial process, ﬁve initial overarching themes were then identiﬁed by the ﬁrst author. These initial themes were further examined individually by the co-authors and following discussion it was agreed that of the ﬁve original themes, three of the themes described similar ﬁndings and were therefore combined.’ (Also presented a table: ‘Relationship between themes identiﬁed in included studies and the meta-ethnographic synthesis themes.’  **Paper S25, pp.3&5 (in appendix 6)**  ‘To translocate study themes between studies, the major themes from each study will be recorded in a grid. These themes will initially be generated from FOI (first order interpretations) or participants views. Comparisons will be made between studies for recurring concepts (which may include similar or disparate findings) and absences of these concepts. Overarching themes that encompass the major findings from all studies will be thus constructed. SOIs will be extracted as author interpretations. TOIs will be generated by combining the FOIs and SOIs across studies.  Stages one and two: coding text and developing descriptive themes – Identifying the ‘findings’ – Line-by-line coding – Developing descriptive themes  • Stage three: generating analytical themes (In the light of the review question)’  ‘5. Translating the studies into each other (aka constructing a common rubric across studies – a form of content analysis – identifying the same themes that are expressed differently) - consider each cell of the grid in turn. Identify the actual key concepts in the paper. Is each concept encompassed by a key concept used to label a row of the grid? Some row key concept titles were taken directly from one paper. Make sure that the key concepts from each individual paper are encompassed by the grid at the end.’  **Paper S25, p.2 (in main manuscript)**  ‘Four key concepts were chosen which reﬂected the main ﬁndings of all included studies. We also abstracted data on standard ﬁelds, such as study aims, design, methods, setting and participants (see online supplementary appendix 3).20 Data were entered into QSR International’s NVivo V.9 software to assist our qualitative analysis and synthesis.21 In step 5, studies were translated into each other by examining the contribution of each study to a key concept. Within the key concepts, similarities and differences in study ﬁndings and contexts were noted, and deviant cases were sought. To address the potential for clinical bias a third reviewer with a non-medical background (SMH) independently read all included articles and cross-checked the derivation and development of the key concepts. In step 6, we synthesised the translations in each key concept to develop third-order interpretations, or higher levels of abstraction of the data for each key concept. We linked the third-order interpretations using a ‘line of argument’, which represented the overarching perspective of GPs towards multimorbidity. The ﬁnal step involved expressing the results of the participants) and second-order interpretations (views of authors). In studies in which GPs were interviewed with another healthcare professional, the analysis was restricted to the views of the GP where possible.  Four key concepts that reﬂected the principal ﬁndings of all included studies were determined. These are reported below and shown in table 2. […] Table 2 Translations between studies with third order interpretation and line of argument formation.  **Paper S32, p.1209.** ‘Data synthesis was rigorous and multi-staged. [..] The ﬁrst phase involved all members of the research team, who engaged in a process of reciprocal translation, whereby over 100 themes and concepts translated onto concept cards (second-order constructs) were examined for convergence (congruent synthesis) and divergence (refutational synthesis) to inductively derive a set of preliminary themes stemming from the data rather than representing apriori categories. First-order constructs were also examined in this way, to ensure that the themes were grounded in the primary data [26]. To add further rigour to the process and explore the validity and sufﬁciency of these preliminary themes, two team members carried out subsequent exercises: ﬁrst-order constructs were content analysed and translated independently, the resulting themes mapped onto the existing third-order constructs, and the second-order constructs were also deductively mapped against the third-order constructs.’ |
| **Reporting of phase 6- the synthesis of translations process**  **Reporting of phase 6- the synthesis of translations process**  **Reporting of phase 6- the synthesis of translations process** | **Paper S5, p. 129.** ‘Translating Studies Into One Another: Third-level constructs were uncovered by comparing second- level constructs across papers, looking for similarities or differences in their descriptions and underlying meanings. Synthesizing the Data: The resulting grid had second-level constructs juxtaposed against third-level constructs. The third-level constructs were the themes that emerged from the second level. These emerging themes were categorized by CC, reviewed by SF, and an initial presentation of these was also offered to a group of Knowledge Users representing a variety of backgrounds including research, frontline care, and long-term care management. Through these processes, the final five overarching themes were decided upon.’  **Paper S6, p. 676.** ‘we synthesized issues from the same row into a common concept by reciprocal translation (step 6). The outcome of this second order analysis was presented as four themes as demonstrated in Table 3. The ﬁnal analysis was negotiated by all authors.’  **Paper S8, p. 150.**  ‘Based on the translated themes, we developed a ‘line-of-argument’ synthesis, which was the most appropriate to depict our understanding of the COPD patients’ experience with PR. The ‘line-of-argument’ is the construction of an interpretation that reveals what is hidden in individualized studies, discovering a whole among a set of parts’  **Paper S12, p. 335.** ‘This phase involves synthesizing the translations into a larger narrative that is greater than what the individual studies would imply. This is an iterative and multilevel process. The synthesized ﬁndings can be used as a basis for improvement efforts and potentially for theory development.’  **Paper S14, pp.3-4.** ‘Finally, we determined how ﬁndings related to each other within and across second-order interpretations (main themes) to produce a tabular display and an overall textual synthesis of immigrant women's experiences of maternity care to serve as a ‘lines-of-argument’ synthesis (Noblit and Hare, 1988) or ‘third-order’ interpretation (Campbell et al., 2011). The ﬁnal stage involved ﬁrst translating studies into each other and then constructing an interpretation that helped discover what was hidden in individual studies to illuminate the overarching synthesis (Noblit and Hare, 1988; Campbell et al., 2011).’  **Paper S16, p. 4**  ‘Synthesizing the (step 3) translation across health topics via interpretive reading of these meta-themes to develop a ‘line of argument’ regarding the process by which schools might influence health. This is presented in the discussion. Through an interpretation of the synthesis, below we present a ‘line of argument’ (step 4 in the meta-ethnography) about how schools might influence health.’  **Paper S18, p. 2.**  ‘In the last phase the themes were synthesized.’  **Paper S19, p. 4**  ‘The reciprocal translations were then brought together by synthesizing them, starting from the identified themes and matching them with their respective quotations. This process involved further re-readings of the original studies, with the final themes obtained being once again compared at the end of the reciprocal translation process.’ | **Paper S10, p. 4.** ‘The secondary key themes of the reciprocal translation were brought together by synthesizing them, starting from the identified secondary key themes and matching them with their respective patients’ quotations of the primary studies. This process involved further re-readings of the original studies. The findings from the translation and the resulting spreadsheet data with secondary themes, explanations, interpretations and subthemes provided the foundation for a third order analysis. In this phase it was possible to re-conceptualize the findings, generating a new interpretation of the secondary-order themes. Each member of the research team independently developed an overarching mind-map and his or her own synthesis model that linked together the translated secondary key themes and authors’ interpretations. These models were merged and discussed. In this phase we also used hand cards to pick apart the original explanations of the authors and subsequently put them together again in clusters. The clusters were compared to each other and classified, resulting in our new third-order concepts with dimensions and subthemes. This process was quite similar to standard primary qualitative research in terms of subjectivity of interpretation, and can be compared to a grounded theory approach that puts the similarities between studies into an interpretive order according to Noblit and Hare a “line of argument”. The line of argument synthesis involved building up a picture of the whole from studies of its parts. Our interpretation aimed to develop a model to explain the overall concepts of patients about individualised medicine’ |
